# The Hypothalamic Epigenetic Landscape in Dietary Obesity

**DOI:** 10.1002/advs.202306379

**Published:** 2023-12-20

**Authors:** Kai Ma, Kaili Yin, Jiong Li, Li Ma, Qun Zhou, Xiyuan Lu, Bo Li, Juxue Li, Gang Wei, Guo Zhang

**Affiliations:** ^1^ Department of Hepatobiliary and Pancreatic Surgery and Zhejiang Provincial Key Laboratory of Pancreatic Disease The First Affiliated Hospital Zhejiang University School of Medicine Hangzhou Zhejiang 310003 China; ^2^ Key Laboratory of Environmental Health Ministry of Education Department of Toxicology School of Public Health Tongji Medical College Huazhong University of Science and Technology Wuhan Hubei 430030 China; ^3^ Institute for Brain Research Collaborative Innovation Center for Brain Science Huazhong University of Science and Technology Wuhan Hubei 430030 China; ^4^ CAS Key Laboratory of Computational Biology Shanghai Institute of Nutrition and Health Shanghai Institutes for Biological Sciences University of Chinese Academy of Sciences (CAS) CAS Shanghai 200031 China; ^5^ State Key Laboratory of Reproductive Medicine Nanjing Medical University Nanjing Jiangsu 211166 China; ^6^ Department of Endocrinology Xinhua Hospital Shanghai Jiao Tong University School of Medicine Shanghai 200092 China; ^7^ Department of Pathophysiology, School of Basic Medical Sciences Henan University Kaifeng Henan 475004 China; ^8^ Institute of Metabolism and Health Henan University Kaifeng Henan China; ^9^ Zhongzhou Laboratory Zhengzhou Henan 450046 China

**Keywords:** chromatin accessibility, dietary obesity, DNA methylation, histone modification, hypothalamus

## Abstract

The hypothalamus in the brain plays a pivotal role in controlling energy balance in vertebrates. Nutritional excess through high‐fat diet (HFD) feeding can dysregulate hypothalamic signaling at multiple levels. Yet, it remains largely unknown in what magnitude HFD feeding may impact epigenetics in this brain region. Here, it is shown that HFD feeding can significantly alter hypothalamic epigenetic events, including posttranslational histone modifications, DNA methylation, and chromatin accessibility. The authors comprehensively analyze the chromatin immunoprecipitation‐sequencing (ChIP‐seq), methylated DNA immunoprecipitation‐sequencing (MeDIP‐seq), single nucleus assay for transposase‐accessible chromatin using sequencing (snATAC‐seq), and RNA‐seq data of the hypothalamus of C57 BL/6 mice fed with a chow or HFD for 1 to 6 months. The chromatins are categorized into 6 states using the obtained ChIP‐seq data for H3K4me3, H3K27ac, H3K9me3, H3K27me3, and H3K36me3. A 1‐month HFD feeding dysregulates histone modifications and DNA methylation more pronouncedly than that of 3‐ or 6‐month. Besides, HFD feeding differentially impacts chromatin accessibility in hypothalamic cells. Thus, the epigenetic landscape is dysregulated in the hypothalamus of dietary obesity mice.

## Graphical abstract

The impact of feeding mice a high‐fat diet on the epigenetic landscape of the hypothalamus is evaluated. A 1‐month HFD feeding exhibits more pronounced dysregulation of histone modifications and DNA methylation than that of 3‐ or 6‐month. HFD feeding differentially impacts chromatin accessibility in hypothalamic cells. Thus, in the hypothalamus of mice with dietary obesity, the epigenetic landscape is dysregulated.

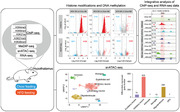

## 1 Introduction

The hypothalamus in the brain plays a fundamental role in controlling energy balance in vertebrates.^[^ ^1^ ^]^ This process involves a number of hypothalamic subregions, including the lateral hypothalamus, ventromedial nucleus, dorsomedial nucleus, paraventricular nucleus, and arcuate nucleus.^[^ ^1^ ^]^ Previous studies have demonstrated that the dysregulated signaling in the hypothalamus contributes to the development of dietary obesity.^[^ ^2^, ^3^, ^4^, ^5^, ^6^ ^]^

The increased epidemic of obesity cannot be explained solely by genetic factor, rather environmental factors are thought to be an important driver. It is presumed that our genes are programmed to store as much fat as possible. Owing to the improvement in living standards, along with the abundance of fast food as well as other energy‐dense diets, our body faces a significant challenge to become accustomed to this new environment. Indeed, the data from bulk RNA sequencing have shown that HFD feeding was able to dramatically impact hypothalamic gene expression.^[^ ^7^, ^8^, ^9^ ^]^

Epigenetics is one of the mechanisms that connects environmental factors to gene activity, and there is an apparent link between the changed diets and the obesity phenotype. Epigenetic events including histone modifications and DNA methylation, are critically involved in transcriptional regulation in eukaryotic cells.^[^ ^10^, ^11^, ^12^, ^13^, ^14^ ^]^ Histone modifications, such as H3K4me3, H3K27ac, H3K9me3, H3K27me3, and H3K36me3, are enriched in specific regions of the genome and are positively or negatively correlated with gene activity. By contrast, 5‐methylcytosine in the promoter region has been mostly correlated with the suppression of gene expression.^[^ ^14^ ^]^ Previous studies showed that the hypothalamic epigenetic pattern changed during animal development as well as other physiological processes.^[^ ^15^, ^16^, ^17^, ^18^, ^19^, ^20^, ^21^, ^22^, ^23^ ^]^ However, a comprehensive analysis remains largely lacking, especially under the condition of dietary obesity. In this study, by leveraging ChIP‐seq, MeDIP‐seq, snATAC‐seq, and RNA‐seq approaches, we aim to fill this knowledge gap.

## 2 Results

### 2.1 Defining Chromatin States in the Mouse Hypothalamus

**Figure 1:**
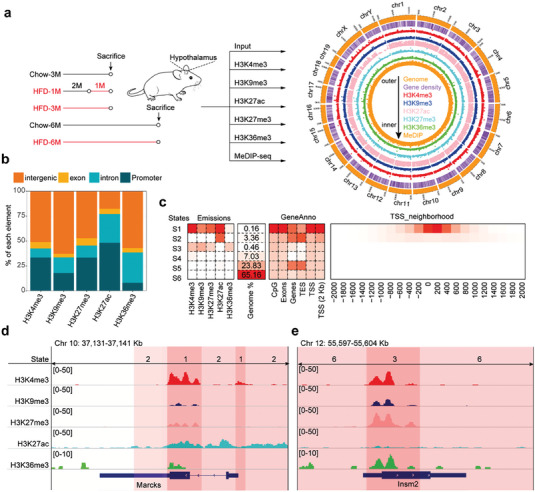
Epigenetic codes shape chromatin states in the mouse hypothalamus. a) Circos plot summarizing the chromosomal distribution of epigenetic marks in the hypothalamus of the 3‐month HFD‐fed mice. The outermost circle depicts the ideograms of each chromosome. The second outermost circle represents gene density, with purple and white indicating high and low density, respectively. Dot plots in other circles indicate the density of epigenetic marks. b) Genomic distribution of histone mark‐enriched regions in the hypothalamus of the 3‐month HFD‐fed mice. The genomic features (exons, introns, and intergenic regions) were defined based on RefSeq (mm10) gene annotations. Promoter was defined as ‐2 kb to +2 kb relative to transcription start site (TSS). c) Emission probabilities of the 6‐state ChromHMM model were calculated from five histone modifications in the hypothalamus of the 3‐month Chow‐fed mice. Each row represents a chromatin state, and each column corresponds to a type of histone modification. The emission parameters were generated from ChIP‐seq data for H3K4me3, H3K9me3, H3K27me3, H3K27ac, and H3K36me3 and represented the enriched possibility, with red and white indicating high and low possibilities, respectively. The heatmap to the right of the emission parameters displays the proportion of the whole genome occupied by each state (Genome %). The heatmap to the right of the genome proportion displays the overlap fold enrichment for various external genomic annotations (GeneAnno). The heatmap to the right of GeneAnno shows the fold enrichment for each state for each 200‐bp bin position within 2 kb around a set of TSSs. A red color corresponds to greater fold enrichment on a column‐specific coloring scale. TES, transcription end site. d, e) Representative genes showing the pattern of occupancy in open regions, such as *Marcks* (states S1 and S2, d), and a bivalent region, such as *Insm2* (state S3, e), in Chow‐3 M mice.

To identify the epigenetic changes, we harvested the hypothalamus of mice fed with a HFD for 1, 3, or 6 months or a chow diet for 3 or 6 months as respective experimental controls (**Figure** **1**a **;** Figure S1, Supporting Information). The hypothalamic tissues were then lysed and used to perform chromatin immunoprecipitation and subsequent DNA sequencing to locate DNA binding sites for histones with posttranslational modifications as well as DNA methylation by methylated DNA immunoprecipitation sequencing (Figure 1a). For the five histone modification marks, histone H3 lysine 4 trimethylation (H3K4me3) is mostly associated with transcriptionally active genes.^[^ ^24^ ^]^ H3 lysine 27 acetylation (H3K27ac) is associated with increased activity of promoter and enhancer regions,^[^ ^25^ ^]^ whereas H3 lysine 9 trimethylation (H3K9me3) corresponds to repressed or silenced loci, such as heterochromatin regions.^[^ ^24^ ^]^ H3 lysine 27 trimethylation (H3K27me3) is associated with Polycomb repression, and H3 lysine 36 trimethylation (H3K36me3) is associated with transcribed regions.^[^ ^25^ ^]^ Initially, we used the Circos plot^[^ ^26^ ^]^ to summarize the chromosomal distribution of epigenetic marks (Figure 1a; Figure S2a, Supporting Information). Among these marks, H3K27ac was localized less to X or Y chromosome, while other marks were distributed uniformly across the chromosomes (Figure 1a; Figure S2a, Supporting Information). Apart from H3K27ac, more than half of the peaks of histone modification marks were localized to intergenic regions (Figure 1b), suggesting a role for distal regions in the regulation of gene activity.^[^ ^27^ ^]^ In addition, for all the histone marks except H3K36me3, the numbers of peaks localized in the promoter region were greater than those in the exon or intron regions (Figure 1b; Figure S2b, Supporting Information).

The chromatins can exist in a variety of conformations. With an understanding of histone modification marks and their associated accessibilities to DNA, we can apply computational analysis with integrative epigenome datasets to segregate chromatin's complexity into discrete number of chromatin states.^[^ ^28^, ^29^, ^30^ ^]^ These states have distinctive enrichments in functional annotations, sequence motifs, and genes that serve shared cellular functions, suggesting discrete biological function.^[^ ^31^ ^]^ Next, we used ChromHMM^[^ ^28^ ^]^ to integrate the five ChIP‐seq datasets to distinguish chromatin states. To do so, we utilized the datasets of 3‐month chow‐fed mice to optimize the parameters of ChromHMM. This analysis allowed us to identify 6 chromatin states (Figure 1c; Figure S3, Supporting Information). Among them, states 1–3 (S1‐S3) defined the active transcription start site (TSS), flanking TSS, and bivalent/poised TSS, respectively. The state S1 was highly enriched for H3K4me3 and H3K27ac, S2 was enriched for H3K27ac, while S3 had enrichments for H3K4me3 and inactive marks H3K9me3 and H3K27me3 (Figure 1c; Figure S3, Supporting Information).

Notably, states S5 and S6 appeared to have less histone modification marks analyzed in this dataset, therefore, they were considered to be the quiescent states (Figure 1c; Figure S3, Supporting Information). For the Chow‐3 M dataset, these two states covered 88.99% of the genome, whereas states representing open/active (S1‐S2) or closed/inactive chromatins (S3‐S4) covered 3.52% or 7.49% of the genome, respectively (Figure 1c). This distribution is comparable to the data reported previously.^[^ ^31^ ^]^ Besides, S1‐S3 in HFD‐1 M, HFD‐3 M, Chow‐6 M, and HFD‐6 M datasets covered 2.29%, 2.55%, 2.47%, 4.21% of the genome, respectively (Figure S3, Supporting Information). And, in HFD‐1 M, HFD‐3 M, Chow‐6 M, and HFD‐6 M datasets, the quiescent states (S5‐S6) covered 97.51%, 97.17%, 89.37% and 95.35% of the genome, respectively (Figure S3, Supporting Information). These data suggest that the coverages of chromatin states varied moderately among treatment groups, and that most of the genomic regions (greater than 80%) are in a less active state in mouse hypothalamus.

The identified open chromatin states suggested that there were actively transcribed genes. To investigate this possibility, we used the genome browser to examine the epigenetic pattern at the *Marcks* locus, a previously identified hypothalamus‐enriched gene involved in the regulation of protein kinase C‐dependent calmodulin signaling.^[^ ^32^, ^33^ ^]^ The *Marcks* locus was enriched by open chromatin marks that characterize S1 and S2 (Figure 1d), which is also consistent with other datasets (Figure S4a,c,e,g, Supporting Information). Meanwhile, S3 defines a bivalent state characterized by both active and inactive histone marks. The insulinoma‐associated 2 (*Insm2*) gene is a member of the Snail transcriptional repressor superfamily.^[^ ^34^, ^35^ ^]^ Insm2 is important for glucose‐stimulated insulin secretion in the pancreatic islet.^[^ ^36^ ^]^ Our data herein show that the *Insm2* gene represents a bivalent state (S3), enriched by H3K4me3, H3K9me3, H3K27me3, and H3K36me3 marks at its locus in the hypothalami of the Chow‐3 M and HFD‐3 M mice (Figure 1e; Figure S4d, Supporting Information). By contrast, the *Insm2* locus in HFD‐1 M was in an active chromatin state (S1) (Figure S4b, Supporting Information). In the Chow‐6 M and HFD‐6 M mice, the *Insm2* gene locus was in an active state but also had H3K27me3 enrichment (Figure S4f,h, Supporting Information).

### 2.2 HFD Feeding Dysregulates Histone Modifications in the Hypothalamus

**Figure 2:**
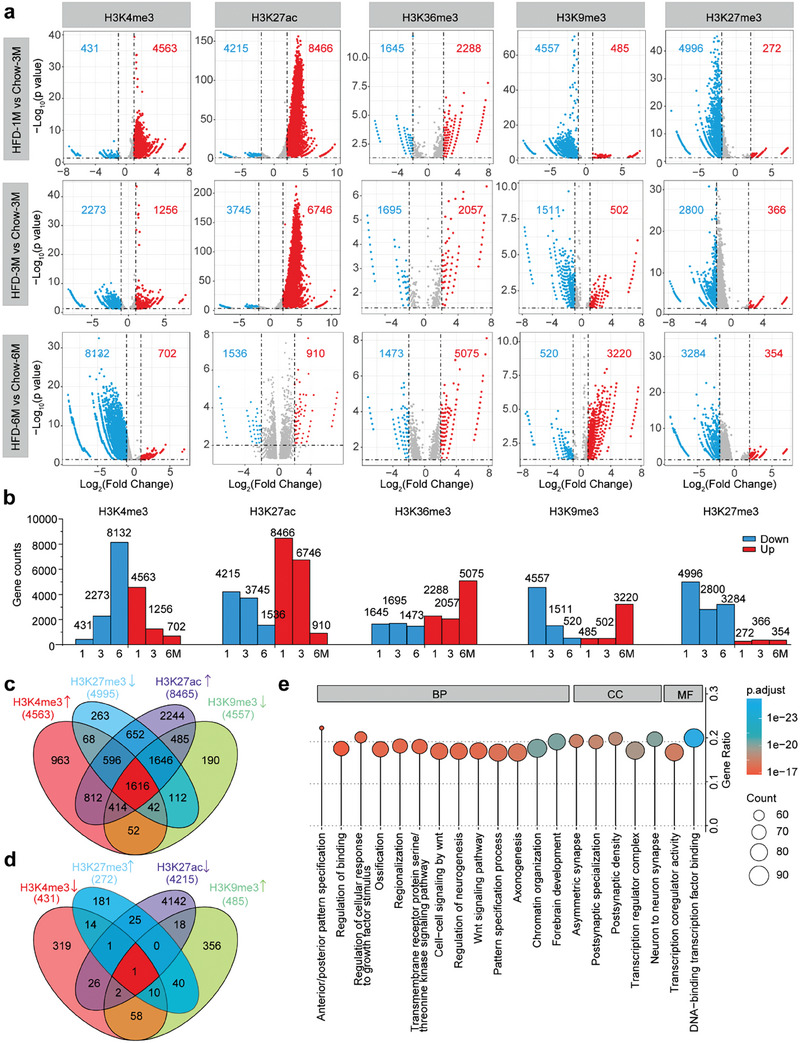
Combined ChIP‐seq analyses reveal the common histone modification changes following HFD feeding. a) Volcano plot displaying the genes in which the promoter regions were differentially occupied by peaks from ChIP‐seq for H3K4me3, H3K9me3, H3K27me3, H3K27ac, and H3K36me3 in the hypothalamus of HFD‐ versus chow‐fed mice. The gene body regions were also included for the H3K36me3 ChIP‐seq data analysis. Red dots indicate significantly more peaks enriched, and blue dots indicate less peaks enriched for each histone mark. Black horizontal dashed lines indicate *p* = 0.05, while *p* = 0.01 for H3K27ac. Black vertical dashed lines indicate the log_2_(fold change) cutoff. The log_2_(fold change) cutoff value for H3K4me3 or H3K9me3 is 1, while for the other three histone marks it is 2. The numbers of genes with differentially enriched histone modifications are shown. b) The numbers of genes with differentially enriched histone modifications. c) Venn diagram showing the number of imputed up‐regulated genes whose promoter regions were enriched with H3K4me3 and H3K27ac, but not with H3K9me3 or H3K27me3 ChIP‐seq peaks in the hypothalamus of 1‐month HFD‐fed mice. d) Venn diagram showing the number of the imputed down‐regulated genes whose promoter regions were not enriched with H3K4me3 and H3K27ac, but with H3K9me3 or H3K27me3 ChIP‐seq peaks in the hypothalamus of 1‐month HFD‐fed mice. e) Lollipop diagram exhibiting the significantly enriched GO terms (top 20 according to *p* value) for genes identified in panel c. BP, biological process; CC, cellular component; MF, molecular function.

Next, we used the MEDIPS tool,^[^ ^37^ ^]^ an R package, to analyze the ChIP‐seq datasets to address whether HFD feeding impacted histone modifications. The enriched peaks associated with different histone marks were identified and annotated using ChIPseeker.^[^ ^38^ ^]^ As opposed to H3K36me3, which is associated with transcribed regions,^[^ ^25^ ^]^ other histone modifications are mostly enriched in the promoter and enhancer regions,^[^ ^24^, ^25^ ^]^ thus the regions annotated as promoter were picked for further analysis. The gene body regions were also included when the H3K36me3 ChIP‐seq data were analyzed. We used volcano plots to show the genes whose promoter regions had enrichment of H3K4me3, H3K9me3, H3K27me3, H3K27ac, or H3K36me3 (**Figure** **2**a). With the same cutoff value, 1‐month HFD treatment significantly impacted histone modifications compared with that of 3‐ or 6‐month treatment (Figure 2a,b). These data suggested that HFD feeding for 1 through 6 months could deregulate histone modifications, yet the inferred changes in global gene expression in the hypothalamus tended to be attenuated following prolonged HFD feeding (Figure 2b), implying that prolonged (6‐month) HFD treatment might trigger an adaptation in histone modifications, e.g., *Nrxn1* (Figure S5, Supporting Information). Nrxn1 is one of the Neurexin family genes, which are central regulators of neural circuits.^[^ ^39^ ^]^ In comparison with 3‐month chow feeding, the epigenetic modifications of *Nrxn1* in the hypothalamus of mice fed a HFD for 1 or 3 months but not 6 months changed dramatically (Figure S5, Supporting Information).

Histone modification is closely related to gene transcription. Next, we imputed the up‐ and down‐regulated genes using the ChIP‐seq datasets (Figure 2c,d; Figure S6a,b, Supporting Information). A 1‐month HFD feeding up‐regulated genes more significantly than the other two diet regimes, while its effect to suppress gene expression was less pronounced (Figure 2c,d; Figure S6a,b, Supporting Information). To investigate the involved pathways, we performed gene ontology (GO) analysis using clusterProfiler.^[^ ^40^ ^]^ These genes were highly enriched in biological processes such as DNA binding and chromatin organization GO terms (Figure 2e). Furthermore, we detected enrichment of genes which were involved in neuronal and forebrain development GO terms (Figure 2e), suggesting that HFD treatment might impact hypothalamic neuronal development.

Next, we identified the common genes whose promoter regions were differentially occupied by peaks from ChIP‐seq of the five histone marks in all three HFD treatment groups. Since H3K36me3 is associated with the transcribed region,^[^ ^25^ ^]^ the peaks in gene body were also included in this dataset. We used Venn diagrams to show the overlap of significantly changed histone modifications (including more or less occupied peaks). The data indicate that almost 1000 genes were significantly altered in the three treatment groups (Figure S7a; Table S1, Supporting Information). Furthermore, GO analysis suggested that the genes were enriched in molecular functions associated with DNA binding transcription factor binding, forebrain development and signaling adaptor activity (Figure S7b; Table S2, Supporting Information).

### 2.3 Trend Analysis of Histone Modifications

**Figure 3:**
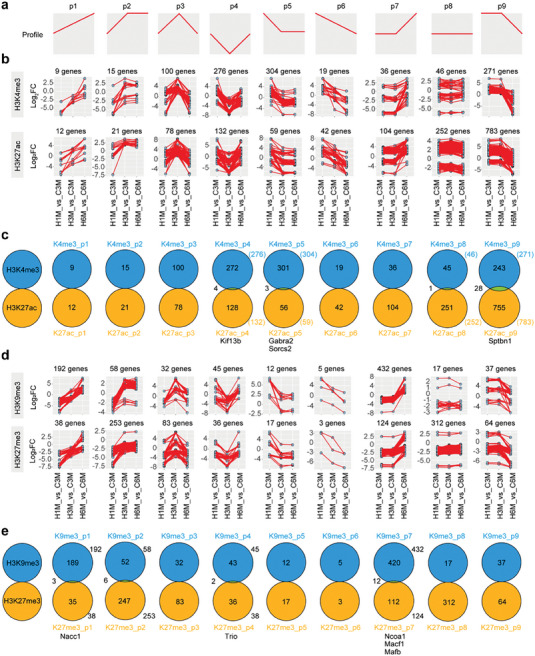
Trend analysis of histone modification profiles after HFD treatment. a) The nine profiles of the histone modification trend change. b) The trend of changes of the active histone modification marks H3K4me3 and H3K27ac. C3M, Chow‐3 M; H1M, HFD‐1 M; H3M, HFD‐3 M; C6M, Chow‐6 M; H6M, HFD‐6 M. c) Venn diagrams displaying the numbers of the overlapping genes between H3K4me3 and H3K27ac. d) The trend of changes of the suppressive histone modification marks H3K9me3 and H3K27me3. e) Venn diagram showing the numbers of the overlapping genes between H3K9me3 and H3K27me3.

Next, we analyzed the trend of the changed histone modifications following HFD treatment. By doing so, we were able to delineate the patterns of the most common changes. This analysis demonstrated that the changes could be clustered into nine profiles (**Figure** **3**a). Subsequently, we integrated the active histone modification marks (H3K4me3 and H3K27ac) as well as the suppressive histone modification marks (H3K9me3 and H3K27me3) to reveal the commonly impacted, post‐translationally modified histone‐associated genes (Figure 3b,d; Figure S8, Supporting Information). The overlapping genes in each profile between two active or two inactive marks were less pronounced (Figure 3c,e). Notably, a subset of the identified genes appeared to be associated with neuronal growth, such as *Kif13b*,^[^ ^41^ ^]^ *Gabra2*,^[^ ^42^ ^]^ *Sorcs2*,^[^ ^43^ ^]^ *Sptbn1*,^[^ ^44^ ^]^ and *Nacc1*,^[^ ^45^ ^]^ and some were associated with the differentiation of brown adipose tissue, such as *Ncoa1* ^[^ ^46^ ^]^ and *Prdm16* ^[^ ^47^ ^]^ (Figure 3c,e; Table S3, Supporting Information). Besides, the distribution of the profiles tended to vary for the four histone modifications (Figure S9, Supporting Information).

### 2.4 HFD Feeding Alters DNA Methylation in the Hypothalamus

**Figure 4:**
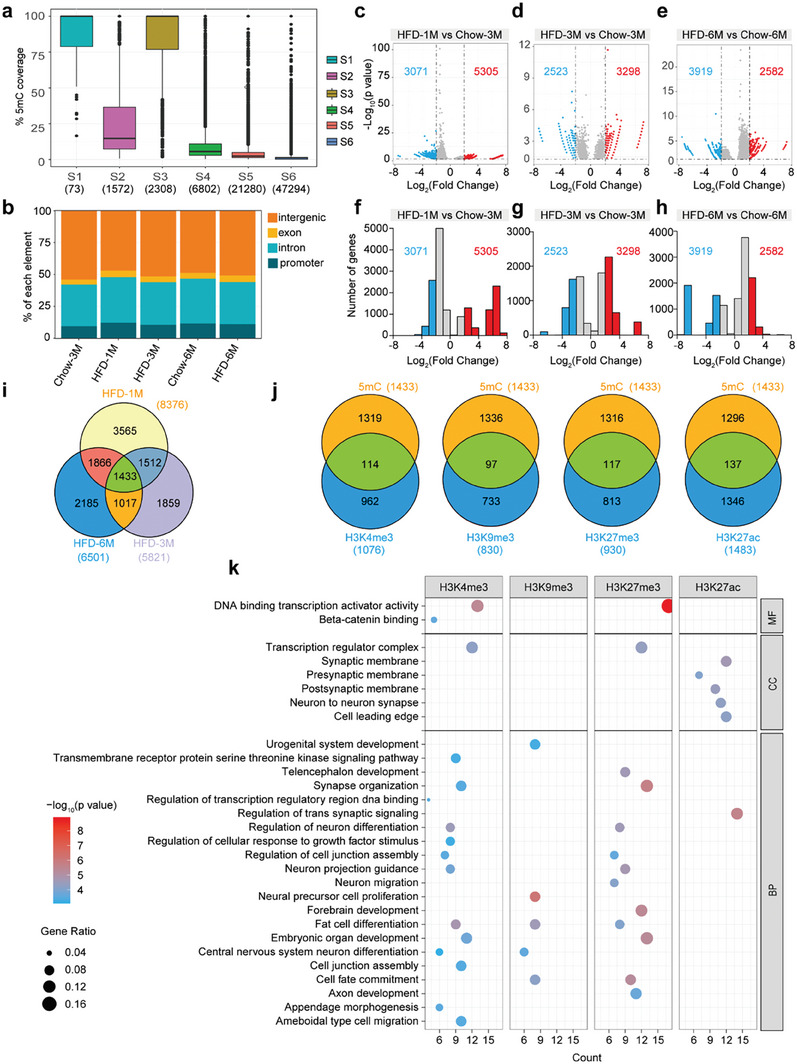
HFD feeding impacts hypothalamic DNA methylation. a) DNA methylation level in each chromatin state. The methylation level of each chromatin state was calculated based on the proportion of each chromatin state region covered by the peaks in MeDIP‐seq for the Chow‐3 M mice. The line in the boxplot represents the median and the boxes represent the first and third quartiles. The whiskers represent data that are within the 1.5× interquartile range. The data beyond the end of the whiskers are outlying points that are plotted individually. The numbers of overlapped regions between the chromatin states and MeDIP‐seq peaks for each state are shown at the bottom. b) Genomic elements associated with DNA methylation. c‐e) Volcano plots showing genes with differentially methylated regions in the hypothalamus of mice fed with HFD. Red dots indicate genes with significantly more methylated DNA fragments enriched, and blue dots indicate genes with less methylated DNA fragments enriched. Black horizontal dashed lines indicate *p* = 0.05, and black vertical dashed lines indicate the log_2_(fold change) cutoff, which is 2. The numbers of genes with more or less methylated DNA fragments are shown. f‐h) Histograms displaying the numbers of genes and their distribution across the range of log_2_(fold change) for the data shown in panels c‐e. i) The overlapping of genes with significantly changed (both more and less methylated fragments) DNA methylation regions is depicted using a Venn diagram. j) The numbers of overlapping genes whose promoter regions had significantly changed occupancy of histone modifications (Figure S7a, Supporting Information) and those with significantly changed pattern of DNA methylation (displayed in panel i). k) Dot plot showing significantly enriched GO terms for genes identified in panel j. The density of color represents the *p* value, and the size of the dots represents the gene ratio. MF, molecular function; CC, cellular component; BP, biological process.

In order to assess the effect of HFD treatment on hypothalamic DNA methylation, we performed MeDIP‐seq. Using these data, we assessed the DNA methylation level of each chromatin state that had been established in our above analysis (Figure 1c). The methylation level of each chromatin state was computed based on the proportion of the genomic region occupied by peaks of MeDIP‐seq relative to that of each chromatin state (**Figure** **4**a; Figure S10, Supporting Information). The data showed that states S1‐S4 had more methylated DNA fragments, except states S2 and S4 in the Chow‐3 M dataset, in which both states had less H3K9me3 and H3K27me3 enriched (Figure 1, 4; Figure S10a–d, Supporting Information). Besides, the states S5 and S6 exhibited low levels of DNA methylation (Figure 4a; Figure S10a–d, Supporting Information).

The MeDIP‐seq peaks were predominantly enriched in intergenic and intronic regions. By contrast, the DNA methylation levels in the promoter and exonic regions were considerably low (Figure 4b). We then analyzed the differentially methylated regions between chow‐ and HFD‐fed mice. Consistent with the ChIP‐seq data, a 1‐month HFD feeding led to more genes with affected DNA methylation regions than that of 3‐ or 6‐month (Figure 4c–h; Table S4, Supporting Information), implicating that extended treatment with HFD also led to an adaptation of DNA methylation in the hypothalamus of mice.

To reveal the commonly deregulated changes in dietary obesity, we used Venn diagrams to illustrate the overlapping genes with differentially methylated regions (Figure 4i). In addition, we analyzed the ChIP‐seq and MeDIP‐seq data to identify the commonly impacted genes due to dietary obesity (Figure 4j; Table S5, Supporting Information). The genes were then subjected to GO analysis. The results showed that these genes were enriched in the GO terms such as DNA binding transcription activator activity, synaptic membrane, and the regulation of neuronal differentiation (Figure 4k).

### 2.5 HFD Feeding Differentially Impacts Chromatin Accessibility in Hypothalamic Cells

**Figure 5:**
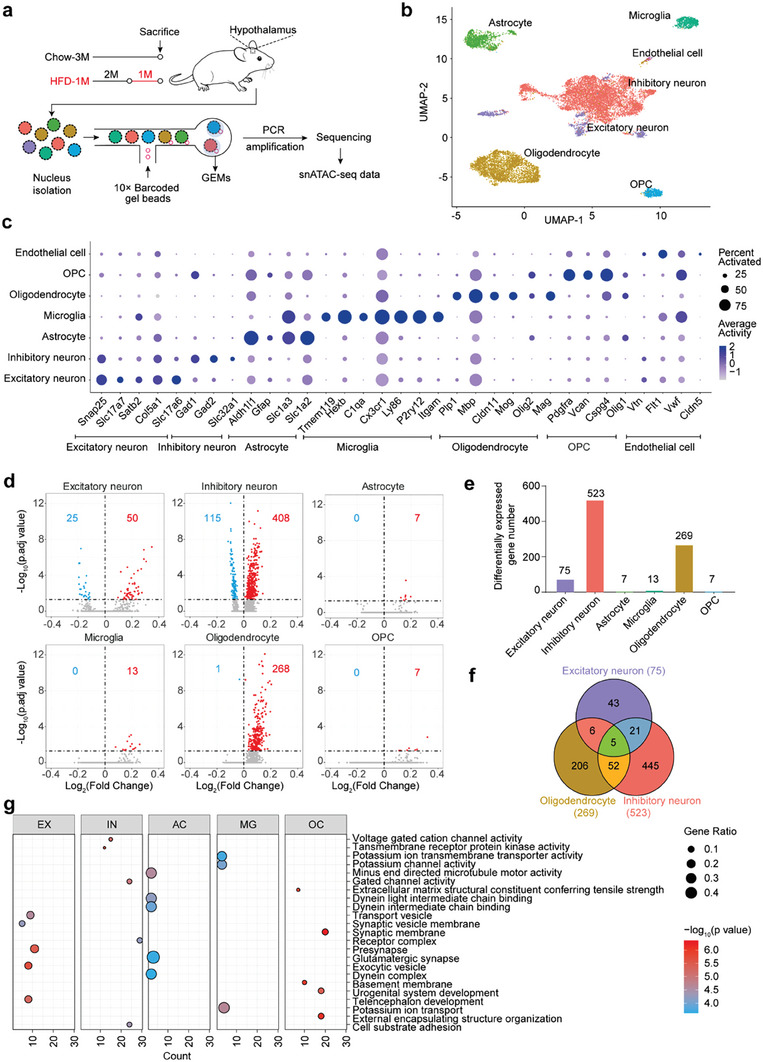
Single nucleus chromatin accessibility analysis of the hypothalamus after a 1‐month HFD feeding. a) Schematic of experimental design. The hypothalamus of Chow‐3 M and HFD‐1 M mice were used to perform snATAC‐seq. GEMs, gel beads in emulsion. b) Uniform manifold approximation and projection (UMAP) plots of snATAC‐seq data colored by cell types. OPC, oligodendrocyte precursor cell. c) Dot plot showing the differential accessibility of cell‐type marker genes. The dot size corresponds to the proportion of cells with detected accessibility of the indicated genes, and the density corresponds to the average accessibility relative to that in all cell types. d) Volcano plots show genes with differential accessibilities. Red and blue colors indicate increased and decreased accessibility, respectively. The horizontal dashed lines indicate *p.adjust* = 0.05. The numbers of genes with differential accessibility are shown. e) The total number of inferred differential genes in each cell type. f) Vern diagram displaying overlapped genes among excitatory neurons, inhibitory neurons, and oligodendrocytes. g) Dot plot showing significantly enriched GO terms (top 5) for differential genes in each cell type. EX, excitatory neuron; IN, inhibitory neuron; AC, astrocyte; MG, microglia; OC, oligodendrocyte.

The hypothalamus is predominantly comprised of neuronal, glial and endothelial cells. It remains unknown whether or not HFD feeding similarly impacts the epigenetics of these cell types. The single‐nucleus assay for transposase‐accessible chromatin using sequencing (snATAC‐seq) is a well‐established approach for detecting open chromatin in individual cell,^[^ ^48^, ^49^, ^50^ ^]^ and the intensities of the snATAC‐seq peak correlated well with active histone marks,^[^ ^51^ ^]^ but barely with H3K27me3,^[^ ^52^ ^]^ a suppressive histone mark. Next, we carried out snATAC‐seq on hypothalamic tissues of mice fed with a chow or a HFD (1 month) using the 10x Genomics Chromium platform (**Figure** **5**a). In total, we obtained 16883 snATAC‐seq datasets. We used Seurat to determine the cellular composition and annotated cells based on marker gene activities (Figure S11a,b, Supporting Information). We generated separate uniform manifold approximation and projection (UMAP) plot for the Chow‐3 M and HFD‐1 M datasets, and then aggregated the UMAP plots for these two experimental groups (Figure 5b; Figure S12a, Supporting Information). Using the snATAC‐seq datasets, we were able to identify all major cell types in the hypothalamus, such as excitatory neuron, inhibitory neuron, astrocyte, microglia, oligodendrocyte, oligodendrocyte precursor cell (OPC), and endothelial cell (Figure 5b), based on the cell type‐specific marker gene activity inferred by chromatin accessibility (Figure 5c).

According to the snATAC‐seq data, the adult mouse hypothalamus consists of 5.9% excitatory neurons, 51% inhibitory neurons, 12.5% astrocytes, 4.4% microglia, 22.2% oligodendrocytes, 3.6% OPCs, and a small number of endothelial cells (Figure S12b, Supporting Information). After HFD feeding, the percentages of various cell types, including inhibitory neurons, OPCs and endothelial cells remained unaltered in the hypothalamus, while the percentages of excitatory neurons and oligodendrocytes were increased by 67.8% and 20.7%, respectively, and the percentage of astrocyte was decreased by 57.6% (Figure S12b, Supporting Information). Subsequently, we compared the gene activity imputed by chromatin accessibility in each cell type between the two dietary regimes. This analysis suggested that a 1‐month HFD feeding dysregulated gene activities in all the cell types except endothelial cells, with inhibitory neurons, oligodendrocytes, and excitatory neurons being the top three impacted cell types (Figure 5d,e; Figure S12c, Supporting Information). The lack of change in endothelial cells was likely owing to the small number of cells assayed (Figure S12, Supporting Information). Furthermore, the activities of a significant fraction of genes were elevated in dietary obesity mice (Figure 5d,e), which was concordant with the imputed gene activity patterns using ChIP‐seq datasets (Figure 2a,b). We compared the lists of impacted genes between two cell types, or among the three most significantly impacted cell types. It appeared that hypothalamic cells were differentially impacted (Figure 5f; Figure S13 and S14, Supporting Information), likely due to their distinctive functions in the control of energy balance as well other physiological processes. We carried out GO analysis of the gene sets (Figure 5g). Differential genes in excitatory neurons were mostly enriched into vesicle‐related GO terms, in inhibitory neurons enriched into voltage‐gated cation channel activity, in astrocytes enriched into dynein‐related GO terms, in microglia enriched into potassium ion channel activity, in oligodendrocytes enriched into synaptic membrane as well as others (Figure 5g).

### 2.6 Transcriptome Changes Following HFD Feeding

**Figure 6:**
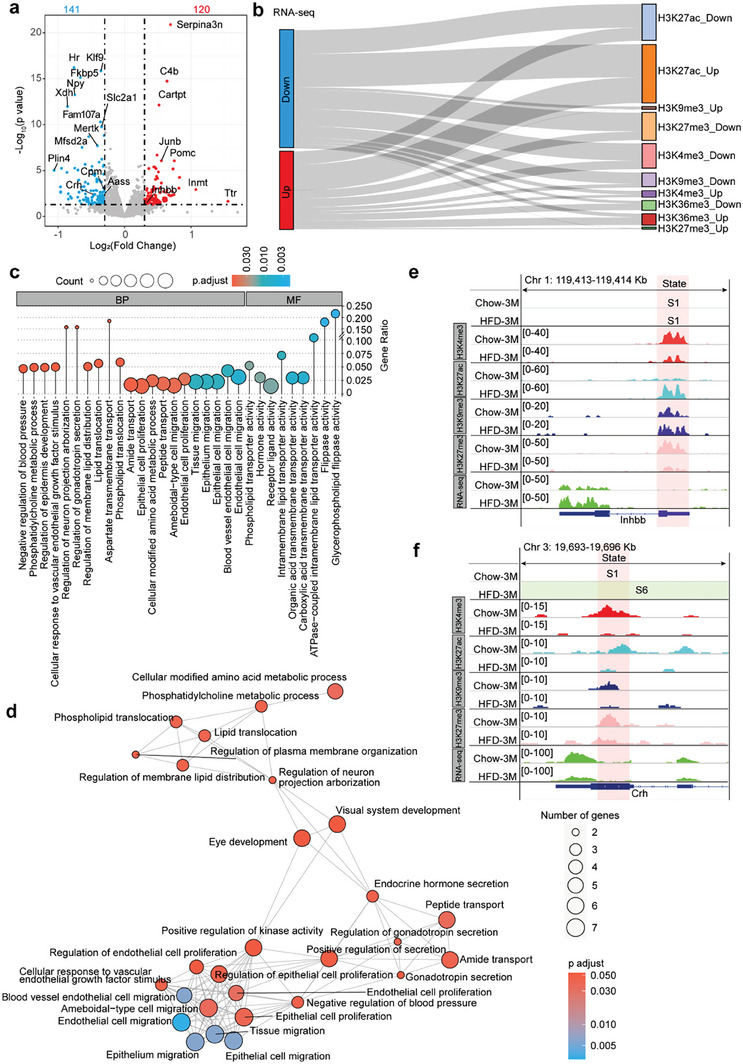
Transcriptomic analysis. a) Volcano plot showing changes in hypothalamic mRNA levels in response to a 3‐month HFD treatment. Red and blue dots indicate significantly up‐ and down‐regulated mRNA levels, respectively. The black horizontal dashed line indicates *p* = 0.05, and the black vertical dashed lines indicate the log_2_(fold change) cutoff, which is 0.3. The numbers of up‐ and down‐regulated genes are shown. The labeled genes are common genes exhibiting overlap with ChIP‐seq data. b) Sankey diagram illustrating the commonly impacted genes in the hypothalamus of 3‐month HFD‐fed mice from RNA‐seq and ChIP‐seq of H3K4me3, H3K9me3, H3K27me3, H3K27ac, and H3K36me3. c) Lollipop diagram to visualize the significantly enriched GO terms for commonly impacted genes revealed in RNA‐seq and ChIP‐seq data analyses. BP, biological process; MF, molecular function. d) Enrichment map networks showing pathways for the common genes identified in panel b. Each node represents a gene set (i.e., a GO term), and each edge represents the overlap between two gene sets. Color indicates the adjusted *p* value, and dot size corresponds to the count. e, f) IGV browser view of *Inhbb* (e) and *Crh* (f) genes in the analyzed datasets.

Next, we performed transcriptomic analysis of the hypothalamus of mice fed a chow or a HFD for 3 months. We were able to obtain 120 up‐ and 141 down‐regulated genes (**Figure** **6**a). These genes were enriched in an array of GO terms, including hormone activity and DNA binding transcription activator activity (Figure S15a, Supporting Information). We then performed network analysis and found that these genes were related to glucose metabolism and cell migration pathways (Figure S15b, Supporting Information). Thereafter, we integrated the RNA‐seq and ChIP‐seq datasets. We intersected the 120 up‐regulated genes with the genes significantly associated with H3K4me3, H3K27ac, and H3K36me3, but not with H3K9me3 or H3K27me3 in the promoter. Besides, we intersected the 141 down‐regulated genes with genes whose promoters demonstrating the reversed pattern of histone modifications (Figure S15c, Supporting Information). The overall results and connections are displayed using the Sankey diagram (Figure 6b). The commonly changed genes were subjected to GO analysis (Figure 6c), and the network was presented (Figure 6d). A subset of genes was enriched for the hormone activity, which had been shown in the ChIP‐seq data (Table S2, Supporting Information). *Inhbb* was one of the genes listed in the GO term of hormone activity. In Chow‐3 M and HFD‐3 M mice, the *Inhbb* promoter was categorized as state S1 (Figure 6e), which was defined as an active state: the highly enriched active mark H3K4me3 with inactive epigenetic marks H3K9me3 and H3K27me3 in Chow‐3 M, as well as the highly enriched active mark H3K27ac with inactive mark H3K9me3 in HFD‐3 M (Figure 6e). The increased H3K27ac and decreased H3K27me3 occupancy at the *Inhbb* promoter region suggested an up‐regulation of *Inhbb* in 3‐month HFD‐fed mice (Figure 6e). Corticotropin‐releasing hormone (CRH) is expressed in the paraventricular nucleus of the hypothalamus.^[^ ^53^ ^]^ Its expression was inhibited in the dietary obesity mice, agreeing with the transition of the *Crh* locus from chromatin state S1 in Chow‐3 M mice to state S6 in HFD‐3 M mice (Figure 6f). The latter was defined as a quiescent state.

## 3 Discussion

Epigenetics is considered “the study of mitotically and/or meiotically heritable changes in gene function that cannot be explained by changes in DNA sequence”^[^ ^54^ ^]^ as opposed to its earlier and broader meaning, “all of the developmental events leading from the fertilized zygote to the mature organism”.^[^ ^55^ ^]^ Over the past three decades, extensive studies have been conducted on epigenetics, and it remains an active topic across almost all biological and biomedical research fields. Among them, studies have demonstrated that epigenetic changes, including histone modification and DNA methylation, are critically involved in the regulation of neurogenesis,^[^ ^56^ ^]^ cognition,^[^ ^57^ ^]^ neurodegeneration,^[^ ^58^ ^]^ as well as other brain function.^[^ ^59^ ^]^ As anticipated, epigenetics plays a key role in the development of the central nervous system.^[^ ^60^ ^]^ This is also true for the hypothalamus, as programmed epigenetic changes have been displayed during animal development.^[^ ^15^, ^16^, ^17^, ^18^, ^19^ ^]^ Previous work also assessed the effects of diet manipulation on the epigenetics of genes involved in energy balance control in the hypothalamus. HFD feeding, for example, led to the hypermethylation of the *Pomc* promoter in male Wistar rats.^[^ ^20^ ^]^ In addition, after a 21‐week HFD treatment, DNA methylation of the *Pomc* promoter appeared to be lower in diet resistant SD rats compared to their obese counterparts.^[^ ^21^ ^]^ A 24‐hour fast, on the other hand, decreased the H3K27me3 level in the chick hypothalamus,^[^ ^22^ ^]^ whereas a 48‐hour fast suppressed H3 acetylation in the ventromedial nucleus of the hypothalamus of C57 BL/6 mice.^[^ ^23^ ^]^ These findings suggest that hypothalamic epigenetic modification plays a significant role in energy balance control. However, a comprehensive study, especially under the circumstance of nutritional excess, remains to be carried out.^[^ ^61^ ^]^ In this work, we show that hypothalamic histone modifications, DNA methylation, and chromatin accessibility can be dysregulated by HFD feeding. Our study thus sheds a new light on the impact of HFD feeding on the hypothalamus, the center of energy balance control in vertebrates.

The changes in hypothalamic H3K27ac and H3K27me3 appeared to be reverted following the prolonged HFD feeding. Indeed, the changes in mRNA levels of a significant fraction of genes can be reverted with prolonged treatment of HFD. For instance, HFD feeding for 1 month led to the up‐regulated expression of 15 out of 53 genes in the liver of male C57 BL/6 mice. These changes were largely diminished in mice fed with a HFD for 12 months compared with chow‐fed controls.^[^ ^62^ ^]^ The authors postulated that this was likely due to the age effect. Besides, in a separate study, Do and colleagues assessed the effect of long‐term HFD feeding on global transcription in the liver of C57 BL/6 mice. They showed that in comparison with short‐term HFD‐fed mice, approximate half of the gene expression changes were reverted after a long‐term HFD treatment.^[^ ^63^ ^]^

A variety of factors may contribute to this reversal, albeit that the underlying causes can be very sophisticated. 1) As stated by Capel et al., age may be a factor leading to diminished gene expression changes following a prolonged HFD feeding.^[^ ^62^ ^]^ 2) In addition, plasma insulin and leptin levels are higher in 12‐month than in 1‐month HFD‐fed mice.^[^ ^62^ ^]^ Hypothalamic cells are known to be responsive to both hormones. Importantly, there exist insulin resistance and leptin resistance in the hypothalamus of mice fed a HFD for an extended period.^[^ ^64^, ^65^ ^]^ These mechanisms may also contribute to the reversal of hypothalamic gene expression changes. 3) In terms of H3K27ac and H3K27me3, changes in enzymes responsible for these two types of histone modification may be related to the reversal after long‐term HFD feeding. There are at least five families of histone acetyltransferase (HAT) in mammalian cells.^[^ ^66^ ^]^ Besides, these HATs form multi‐protein complexes. It is known that the enhancer of zeste 2 polycomb repressive complex 2 subunit (EZH2), the functional enzymatic component of polycomb repressor complex 2 (PRC2), is responsible for the trimethylation of H3 at lysine 27. Therefore, changes in gene expression and/or enzyme activity might consequently lead to changes in H3K27ac or H3K27me3 modification in hypothalamic cells over prolonged HFD treatment. Nonetheless, our data demonstrate that both the numbers of genes with increased and decreased H3K27ac modifications at 1 month of HFD feeding were reverted following chronic HFD treatment. This suggests that the underlying causes can be even more complicated.

As two crucial components of the integrative system for energy balance regulation, the hypothalamus and the adipose tissues are known to be tightly coupled, and there exists crosstalk between them. In particular, the seminal work by Dr. Bartness and colleagues uncovered the sympathetic nervous system (SNS) innervation of adipose tissues. Using a fluorescent tracer, they showed that the white adipose tissue (WAT) is innervated by the postganglionic neurons of the SNS.^[^ ^67^ ^]^ Furthermore, their work identified the neural axis, including the hypothalamus, that ultimately innervates the WATs in rodents using viral tracing approaches. In a separate study using the same approach, Dr. Friedman and colleagues showed that neurons in the paraventricular nucleus, the lateral hypothalamus, and the arcuate nucleus could indirectly project to epididymal WAT.^[^ ^68^ ^]^ In addition, leptin regulates the SNS innervation of WAT via the neurons in the arcuate nucleus.^[^ ^69^ ^]^

Previous studies have also demonstrated that the manipulations in the hypothalamus led to changes in gene expression in WATs. For example, gene transfer of BDNF in the mediobasal hypothalamus could remodel adipose tissues and alter gene expression.^[^ ^70^ ^]^ Loss of RIIβ, a subunit of protein kinase A, promoted browning and impacted gene expression in inguinal WAT (iWAT). Re‐expression of RIIβ in the dorsomedial nucleus of the hypothalamus could abrogate WAT browning and restore gene expression in the iWAT.^[^ ^71^ ^]^ These findings suggest that the hypothalamus might regulate epigenetic events in the WAT. Since our current study shows that HFD feeding leads to changes in histone modifications and DNA methylation, it is likely that hypothalamic epigenetic changes may lead to epigenetic alteration in the WAT. This possible crosstalk warrants further investigation.

As mentioned above, previous studies have interrogated the effects of diet manipulations on the methylation of specific genes, e.g., *Pomc*,^[^ ^20^, ^21^ ^]^ or on the global H3K27me3 level^[^ ^22^ ^]^ or H3 acetylation level^[^ ^23^ ^]^ in the hypothalamus following fast. A comprehensive, multi‐omic analysis focusing on hypothalamic epigenetic changes under dietary obesity, remains lacking. In the current study, we carried out combinatorial analysis of the ChIP‐seq, MeDIP‐seq, snATAC‐seq, and RNA‐seq data of the hypothalami of mice fed with chow or HFD for various periods. We showed that 1 month of HFD feeding deregulated hypothalamic histone modifications and DNA methylation more significantly than that of 3 or 6 months. Importantly, the data generated in this work can aid studies of hypothalamic epigenetic events in the development of dietary obesity.

The tissue in the hypothalamus, as with those in other brain regions, is heterogeneous. Single cell RNA sequencing of hypothalamic sub‐regions of diet‐induced obesity mice have been carried out.^[^ ^72^, ^73^, ^74^ ^]^ Those studies uncovered gene expression changes in a cell‐specific manner.^[^ ^72^, ^73^, ^74^ ^]^ Yet, it remains to be established for the obesity‐related, cell‐specific epigenetic changes in the hypothalamus. In the current study, we performed snATAC‐seq of the hypothalami of both chow‐ and HFD‐fed mice. This analysis demonstrates that the inhibitory neurons, oligodendrocytes, and excitatory neurons are the most significantly impacted cell types by a 4‐week HFD feeding. These data suggest that HFD can deregulate chromatin accessibility in a cell type‐specific manner in the hypothalamus.

We acknowledge limitations of the current study. As stated above, the hypothalamic tissue is heterogeneous. We did not analyze the changes in histone modifications or DNA methylation in single cell resolution, which are pivotal for understanding the impact of dietary obesity on individual hypothalamic cell types. In addition, the multi‐omic approach employed in the current study unveiled genes whose expression were dysregulated by HFD feedings. We did not investigate the underlying cause(s). Besides, it remains to be established whether these genes are relevant in the development of dietary obesity.

## 4 Conclusion

In summary, we show that HFD feeding can dysregulate histone modifications, DNA methylation, and chromatin accessibility across the genome in the hypothalamus. A short‐term HFD feeding appears to more significantly disturb epigenetic landscape than that of long‐term one, and appears to dysregulate chromatin accessibility in a cell type‐specific fashion. Overall, our study highlights a profound effect of HFD feeding on hypothalamic epigenetic landscape. The relevance of this alteration in the pathophysiology of obesity and its related co‐morbidities remains to be determined.

## 5 Experimental Section

### Animal Procedures

Male C57 BL/6 mice were purchased from Vital River Laboratory Animal Technology (Beijing, China). Temperature (22‐24 °C), humidity, and illumination (12 h light/12 h dark cycle) were controlled, and mice had free access to diets and water. For 3‐ or 6‐month HFD treatment, mice were placed on the HFD at 6 to 7 weeks of age. For a 1‐month HFD treatment, mice were fed HFD at 14 to 15 weeks of age. All experimental procedures were approved by the IACUC at the Huazhong University of Science and Technology. HFD (60% kcal from fat) and rodent chow (9.4% kcal from fat) were purchased from Medicience (Yangzhou, China) and HFK Bioscience (Beijing, China), respectively. The body weights of mice were regularly measured.

### Genomic DNA and RNA Extractions

Genomic DNA was extracted from the hypothalamus of male C57 BL/6 mice fed with chow or HFD for 1, 3, and 6 months using the DNeasy kit (Qiagen, Germantown, MD). Total RNAs were extracted from the hypothalamus of chow‐ or HFD‐fed mice using the RNeasy Mini Kit (Qiagen, Germantown, MD) according to the manufacturer's instructions.

### ChIP‐Sequencing and Data Processing

Hypothalamic tissues from more than 10 mice were combined and homogenized with a Dounce tissue homogenizer. Subsequent cross‐linking, chromatin isolation, sonication, immunoprecipitation, and DNA purification were carried out using an EZ‐Magna ChIP A/G Chromatin Immunoprecipitation kit (Millipore, Burlington, MA). The antibodies used for immunoprecipitation were anti‐H3K4me3 (Cat# 04–745, Millipore), anti‐H3K9me3 (Cat# ab8898, Abcam, Waltham, MA), anti‐H3K27me3 (Cat# 9733, Cell Signaling Technologies, Danvers, MA), anti‐H3K27ac (Cat# ab4729, Abcam), and anti‐H3K36me3 (Cat# ab9050, Abcam). The prepared libraries were sequenced on the HiSeq2500 v4 for 100 bp paired‐end reads at Berry Genomics (Beijing, China). After sequencing, reads that passed quality trimming were aligned against the mouse reference genome (mm10) using Bowtie2. The resulting BAM files were then processed through Bedtools (to clean, deduplicate and sort) for downstream analysis and visualization. For ChIP‐seq datasets, peak calling was performed using Sicer.^[^ ^75^ ^]^ All mapped BAM files were converted to Bigwig using Samtools for visualization in IGV (Integrative Genomics Viewer).

### MeDIP‐Sequencing and Data Processing

The MeDIP was performed using a MagMeDIP kit (Diagenode, Denville, NJ) according to the manufacturer's instructions. MeDIP libraries were sequenced on Illumina HiSeq2500 at Shanghai Biotechnology Corporation (Shanghai, China). After sequencing, 50 bp single‐end reads that passed quality trimming were aligned against the mouse reference genome (mm10) using Bowtie2. For MeDIP‐seq datasets, peak calling was performed using Macs2.^[^ ^76^ ^]^ Bedtools was used to intersect the peak regions found by Macs2 with chromatin state regions. We calculated the proportion of each chromatin state region covered by MeDIP‐seq peaks.

### ChIP‐Seq and MeDIP‐Seq Data Analysis and Visualization

To assess the combinatorial patterns of five chromatin marks, chromatin states were obtained from aligned binarized BAM files using the LearnModel command in ChromHMM (ver. 1.23),^[^ ^28^ ^]^ where the number of states was set to 6 after optimization. The peaks were used to generate circular diagrams using shinyCircos.^[^ ^26^ ^]^ To plot ChIP‐seq signals with mapped reads, we used deepTools to generate bigwig files, normalized with reads per kilobase per million mapped reads (RPKM) for genome browser view. For both ChIP‐seq and MeDIP‐seq data, we used the MEDIPS tool,^[^ ^37^ ^]^ an R package, to detect significant enrichment of the signal of HFD feeding relative to chow feeding groups with a raw *p* value < 0.05. The MEDIPS analysis used edgeR^[^ ^77^ ^]^ method to calculate differential coverage using the exact test, which is based on the quantile‐adjusted conditional maximum likelihood method,^[^ ^77^ ^]^ and Bonferroni was used to perform the multiple testing correction. Chromosomal positions (peaks) were customized within an annotation package from Bioconductor, *TxDb.Mmusculus.UCSC.mm10.knownGene*: Annotation package for TxDb object(s) in R package ChIPseeker (ver. 1.30.3).^[^ ^38^ ^]^ Gene ontology analysis was implemented with the R package ClusterProfiler.^[^ ^40^ ^]^ Venn diagrams were generated with the Vennerable and VennDiagram R packages. Volcano and box plots were generated with the ggplot2 R package. The log_2_(fold change) cutoff value for H3K4me3 or H3K9me3 ChIP‐seq was set at 1, while other three histone marks and MeDIP‐seq were set at 2. We used the networkD3 R package to generate Sankey diagram.

The trend analysis was performed according to the value of log_2_(fold change) using ChIP‐seq data. A slope indicated the difference of log_2_(fold change) of a gene between two adjacent groups (e.g., HFD‐3 M versus HFD‐1 M) was equal to or greater than 1, and a horizontal line meant the difference of log_2_(fold change) was less than 1. For example, the profile p1 indicated the change of the ChIP‐seq peaks in the promoter and gene body (H3K36me3) was from less to more in the three time points.

### Single‐Nucleus ATAC‐Sequencing and Data Analysis—Nuclei Preparation

Single nuclei from mouse hypothalamus were isolated using the reagents and the nuclei isolation for single cell ATAC sequencing protocol from 10x Genomics (Pleasanton, CA). An appropriate volume of chilled diluted nuclei buffer (Cat# 2 000 207, 10x Genomics) was used to resuspend cell nuclei, which were then counted.

### Single‐Nucleus ATAC‐Sequencing and Data Analysis—snATAC‐Seq

Resuspended cell nuclei were used for transposition and loaded into the Chromium Next GEM Chip H with 10× reagents and barcoded with a 10× Chromium Controller (10x Genomics). DNA fragments from the barcoded cells were subsequently amplified, and sequencing libraries were constructed with reagents from a Chromium NextGEM Single Cell ATAC Reagent kit ver 2 (Cat# CG000496, 10x Genomics) according to the manufacturer's instructions. Libraries were then pooled and loaded on an Illumina NovaSeq 6000 PE50, a 50 bp paired‐end module at Novogene (Beijing, China).

### Single‐Nucleus ATAC‐Sequencing and Data Analysis—Data Preprocessing

The computational pipeline for processing the data produced from the Chromium Single Cell ATAC Solution was according to Cell Ranger ATAC Algorithms Overview (https://support.10xgenomics.com/single‐cell‐atac/software/pipelines/latest/algorithms/overview). Briefly, raw sequencing data was converted to fastq format using cellranger‐atac mkfastq. snATAC‐seq reads that passed quality control were aligned to the reference genome (mm10) and quantified using cellranger‐atac count (10x Genomics) using default parameters. Barcode sequences were obtained from the I5 index reads. Then, the cutadapt tool was used to identify and trim any adapter sequence in each read. Third, the trimmed read pairs were aligned to a reference using BWA‐MEM with default parameters. Reads less than 25 bp were not aligned and flagged as unmapped. Fragments were identified as read pairs with MAPQ > 30, non‐mitochondria read, and not chimerically mapped. The start and end of the fragments were adjusted (+4 for the + strand and −5 for the – strand) to account for the 9‐bp region the transposase enzyme occupies during the transposition.

### Single‐Nucleus ATAC‐Sequencing and Data Analysis—Peak Annotation

Cell Ranger ATAC used bedtools closest ‐D = b to associate each peak with genes based on the closest transcription start sites (packaged within the reference) such that the peak was within 1000 bases upstream or 100 bases downstream of the TSS. Additionally, Cell Ranger ATAC also associated genes with putative distal peaks that were much further from the TSS and were less than 100 kb upstream or downstream from the ends of the transcript. Peak genomic annotation is generated using ChIPseeker (ver. 1.5.1).^[^ ^38^ ^]^

### Single‐Nucleus ATAC‐Sequencing and Data Analysis—Clustering and Differential Accessibility Analysis

The peak‐by‐cell data matrix was processed using the Signac (ver. 1.3.0). A quality control cutoff of a minimum of 200 fragments per cell in the peak region, a maximum of 95% quantile fragments per cell in the peak region, a maximum of 4 nucleosome signal value, and a minimum of 2 transcription start sites enrichment were applied to trim the dataset of low‐quality cells. Then, the doublet cells were removed by ArchR (ver. 1.0.1). Next, Signac performed term frequency‐inverse document frequency (TF‐IDF) normalization, and the variable features (top 25%) were used to perform latent semantic indexing (LSI), and the first 30 components were calculated. These components were then used to generate a uniform manifold approximation and projection dimensionality reduction. Post UMAP, a shared‐nearest‐neighbor graph was used to cluster the cells via Seurat's Louvain algorithm. Differentially accessible peaks of each cluster were generated using Seurat's default FindAllMarkers() function. Pseudobulk profiles by cluster highlighting fragment stack ups at particular genomic regions were generated using Signac. In addition, the gene expression level was inferred using the GeneActivity() function.

### Single‐Nucleus ATAC‐Sequencing and Data Analysis—Enrichment Analysis

Genes related to differential peaks were identified by *p.adjust* <0.05. Gene ontology enrichment analysis of genes was implemented by the clusterProfiler^[^ ^40^ ^]^ R package, in which gene length bias was corrected. GO terms with an adjusted *p* value less than 0.05 were considered significantly enriched.

### RNA Sequencing and Analysis

RNA‐seq libraries were constructed using 1 µg of total RNA, and then were sequenced on the Illumina HiSeq 2500 platform, generating 100‐bp paired‐end reads. Sequencing quality was assessed using FastQC. The reads that passed quality control were aligned to the reference genome (mm10) by using STAR (ver. 2.7.8a). Differential expression analysis of RNA‐seq data was performed as described in our previous study.^[^ ^78^ ^]^

### Statistics

Body weight data were analyzed using two‐tailed Student's *t*‐test. Unless otherwise noted, a *p* value less than 0.05 was considered statistically significant.

## Conflict of Interest

The authors declare no conflict of interest.

## Author Contributions

K.M. and K.Y. contributed equally to this work. K.M. analyzed the data and drafted the paper. K.Y., J.L., Q.Z., and X.L. carried out animal experiments and prepared ChIP‐seq and snATAC‐seq samples. L.M. and G.W. analyzed the data. B.L. and J.L. provided biological samples. G.Z. conceived the study, designed the experiments, and edited the paper. All of the authors commented on the paper.

## Supporting information

Supporting Information

Supplemental Table 1

Supplemental Table 2

Supplemental Table 3

Supplemental Table 4

Supplemental Table 5

## Data Availability

The raw sequence data reported in this paper have been deposited in the Genome Sequence Archive^[^ ^79^ ^]^ in the National Genomics Data Center,^[^ ^80^ ^]^ China National Center for Bioinformation/Beijing Institute of Genomics, Chinese Academy of Sciences (GSA CRA009776 for ChIP‐seq, MeDIP‐seq, and snATAC‐seq data; GSA CRA002561 for RNA‐seq data). These data are publicly accessible at https://ngdc.cncb.ac.cn/gsa. Bedfiles of ChIP‐seq and MeDIP‐seq have been deposited in the OMIX database under the accession no. OMIX002960 (https://ngdc.cncb.ac.cn/omix).
