# Supplementary material for: The Hypothalamic Epigenetic Landscape in Dietary Obesity
Source: Adv Sci (Weinh). 2023 Dec 20;11(9):2306379. doi: 10.1002/advs.202306379 (PMC10916675; doi:10.1002/advs.202306379)
Supplement: Supplementary file 1 — Supporting Information [file ADVS-11-2306379-s001.pdf]

ADVANCED SCIENCE
Open Access

Supporting Information

for *Adv. Sci.*, DOI 10.1002/advs.202306379

The Hypothalamic Epigenetic Landscape in Dietary Obesity

*Kai Ma, Kaili Yin, Jiong Li, Li Ma, Qun Zhou, Xiyuan Lu, Bo Li, Juxue Li, Gang Wei and Guo Zhang**

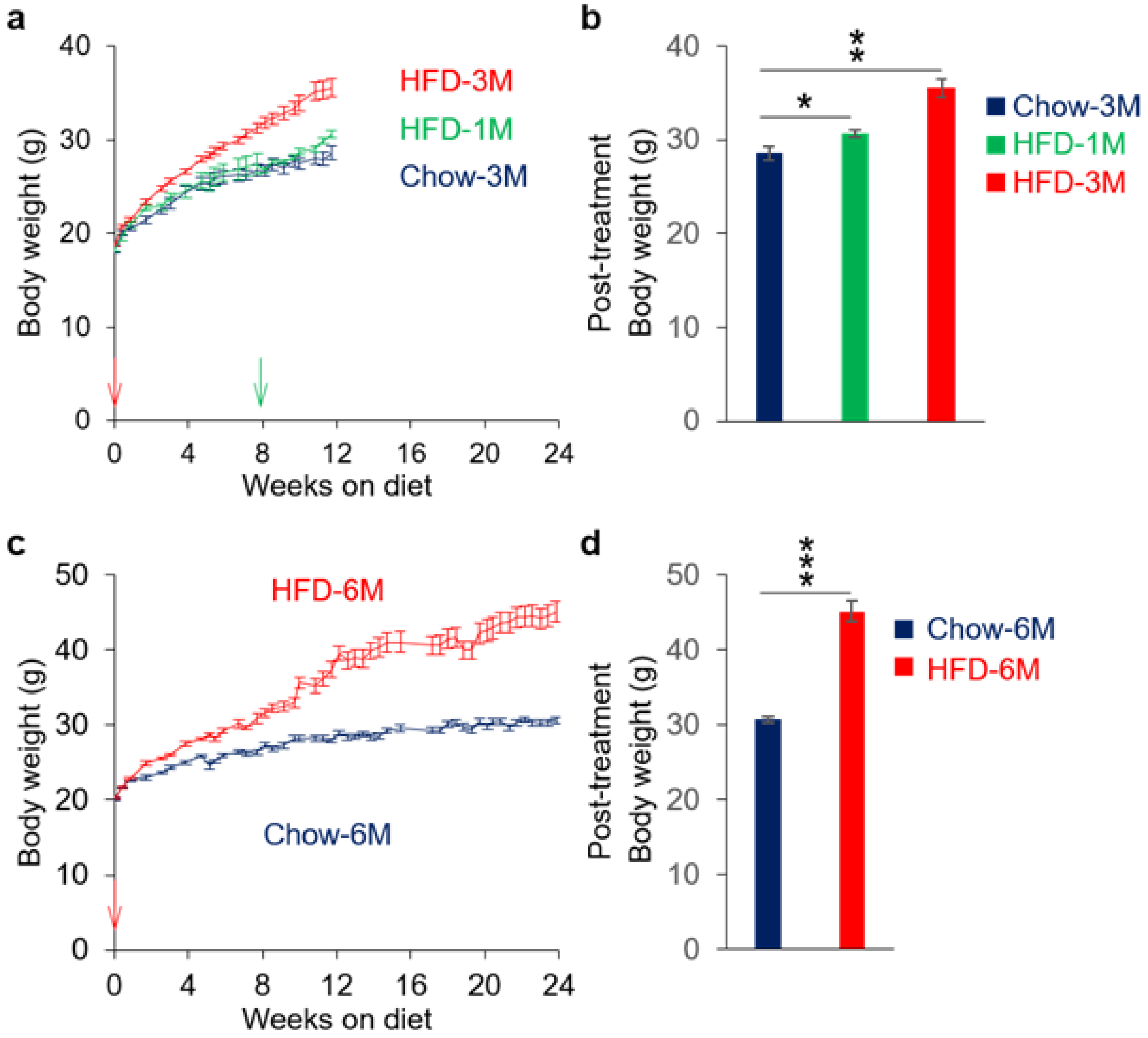

Figure S1. Body weight of mice. a) Body weight of male C57 BL/6 mice maintained on a chow diet or fed a HFD for 1 or 3 months. Arrows indicate the beginnings of HFD feeding. n = 9 (Chow-3M), 10 (HFD-1M), or 21 (HFD-3M). b) The post-treatment body weight of mice. n = 9 (Chow-3M), 10 (HFD-1M), or 21 (HFD-3M). c) Body weight of male C57 BL/6 mice maintained on a chow diet or fed a HFD for 6 months. Arrow indicates the beginning of HFD feeding. n = 16 (Chow-6M), or 21 (HFD-6M). d) The post-treatment body weight of mice. n = 16 (Chow-6M), or 21 (HFD-6M). Data are presented as mean ± SEM. *p<0.05, **p<0.01, ***p<0.001, two-tailed Student's t-test.

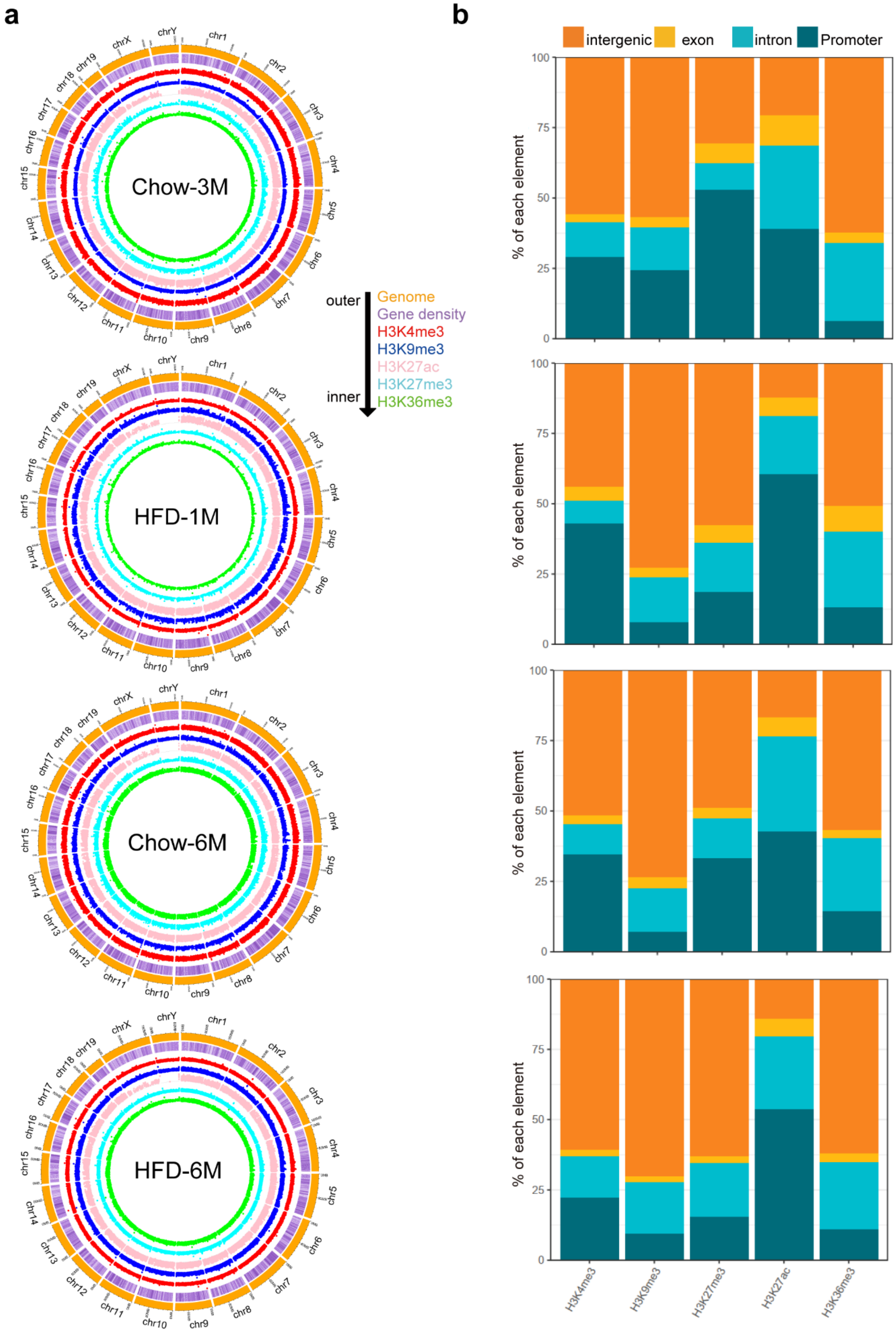

a
b
Chow-3M
HFD-1M
Chow-6M
HFD-6M
chrY
chr1
chr2
chr3
chr4
chr5
chr6
chr7
chr8
chr9
chr10
chr11
chr12
chr13
chr14
chr15
chr16
chr17
chr18
chr19
chrX
outer
inner
Genome
Gene density
H3K4me3
H3K9me3
H3K27ac
H3K27me3
H3K36me3
intergenic
exon
intron
Promoter
% of each element
100
75
50
25
0
H3K4me3
H3K9me3
H3K27me3
H3K27ac
H3K36me3

Figure S2. Summary of the chromosomal distribution of epigenetic marks. a) Circos plots summarizing the chromosomal distribution of epigenetic marks. The outermost circle depicts the ideograms of each chromosome. The second outermost circle represents gene density, with purple and white indicating high and low density, respectively. Dot plots in other circles display the density of epigenetic marks, from outer to inner: H3K4me3, H3K9me3, H3K27ac, H3K27me3, H3K36me3. b) Relative proportion of annotated genomic elements for each histone modification.

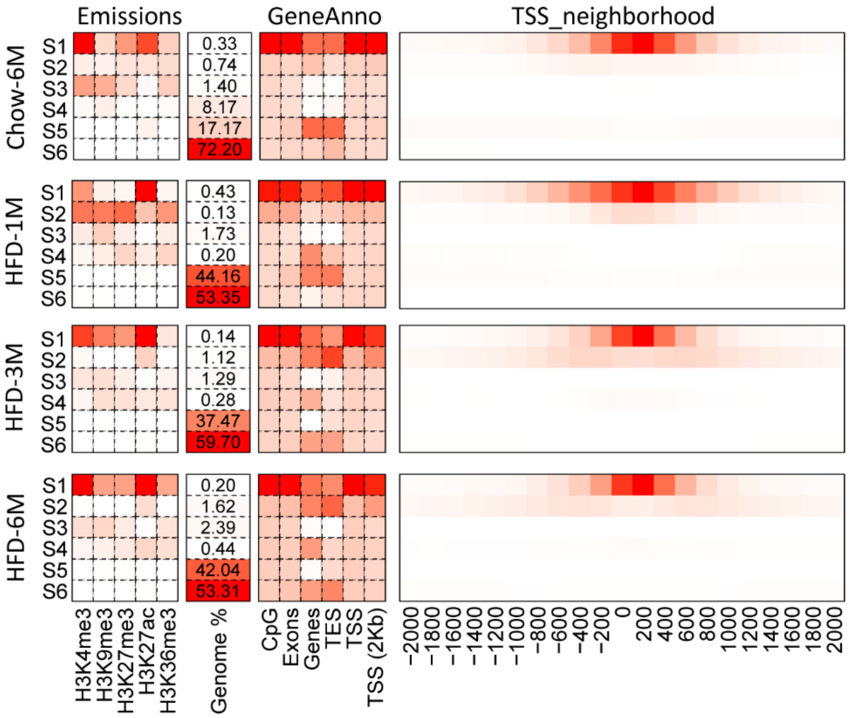

Figure S3. Hypothalamic chromatin state following diet treatments. Emission probabilities of the 6-states ChromHMM model were calculated from five histone modifications in the hypothalamus of mice fed with chow for 6 months of HFD for 1, 3, or 6 months. Each row represents a chromatin state, and each column corresponds to a histone modification. The emission parameters were generated from ChIP-seq data for H3K4me3, H3K9me3, H3K27me3, H3K27ac, and H3K36me3, and represent the enrichment possibility, with red and white indicating a high and low possibility, respectively. The heatmap to the right of the emission parameters displays the proportion of the whole genome occupied by each state (Genome %). The heatmap to the right of the genome proportion displays the overlap fold enrichment for various external genomic annotations (GeneAnno). TES, transcription end site. The heatmap to the right of GeneAnno shows the fold enrichment for each state for each 200-bp bin position within 2 kb around a set of transcription start sites (TSSs). A red color corresponds to a greater fold enrichment on a column-specific coloring scale.

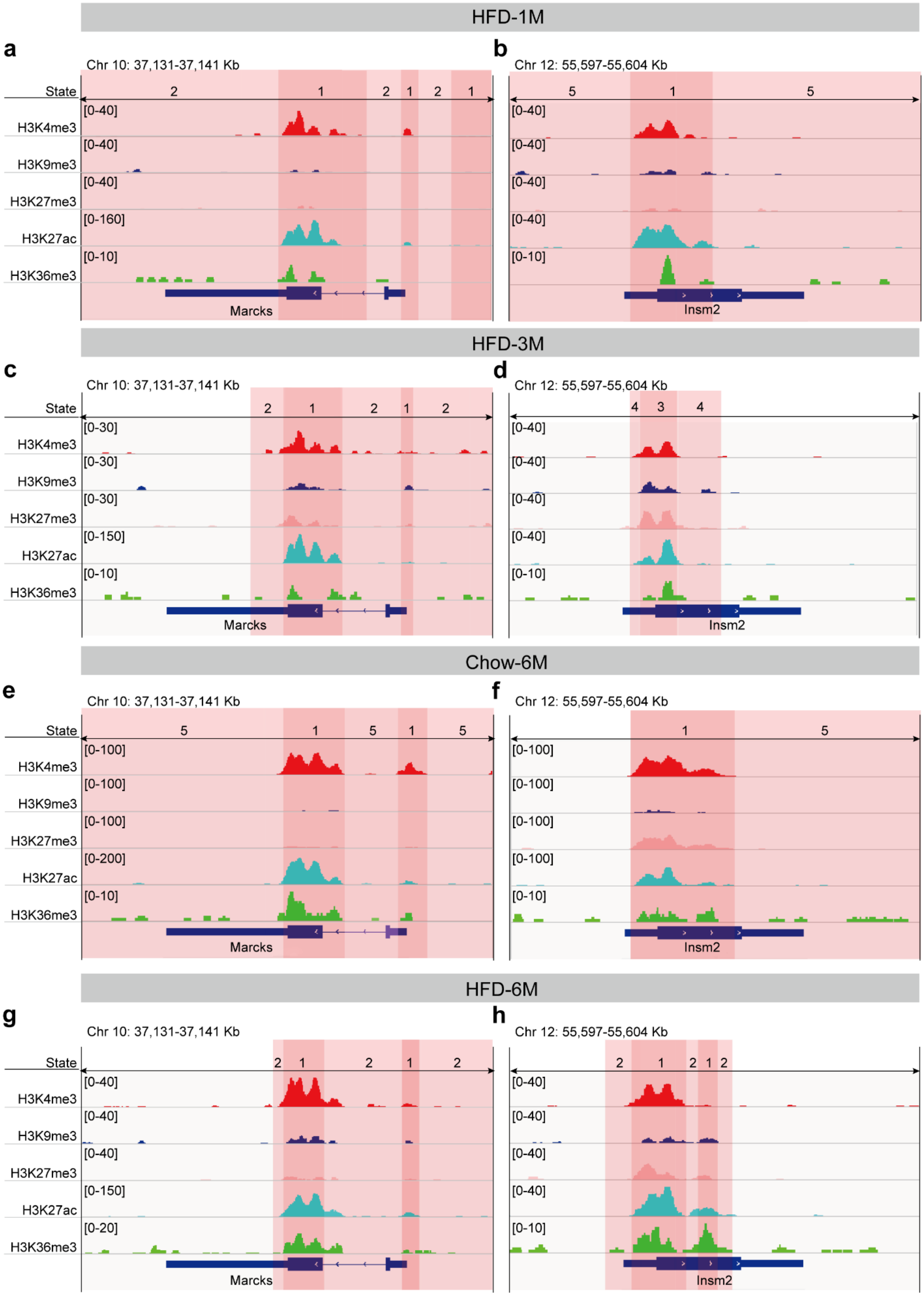

HFD-1M
a
Chr 10: 37,131-37,141 Kb
State
2
1
2
1
2
1
[0-40]
H3K4me3
[0-40]
H3K9me3
[0-40]
H3K27me3
[0-160]
H3K27ac
[0-10]
H3K36me3
Marcks
b
Chr 12: 55,597-55,604 Kb
5
1
5
[0-40]
[0-40]
[0-40]
[0-40]
[0-10]
Insm2
HFD-3M
c
Chr 10: 37,131-37,141 Kb
State
2
1
2
1
2
[0-30]
H3K4me3
[0-30]
H3K9me3
[0-30]
H3K27me3
[0-150]
H3K27ac
[0-10]
H3K36me3
Marcks
d
Chr 12: 55,597-55,604 Kb
4
3
4
[0-40]
[0-40]
[0-40]
[0-40]
[0-10]
Insm2
Chow-6M
e
Chr 10: 37,131-37,141 Kb
State
5
1
5
1
5
[0-100]
H3K4me3
[0-100]
H3K9me3
[0-100]
H3K27me3
[0-200]
H3K27ac
[0-10]
H3K36me3
Marcks
f
Chr 12: 55,597-55,604 Kb
1
5
[0-100]
[0-100]
[0-100]
[0-100]
[0-10]
Insm2
HFD-6M
g
Chr 10: 37,131-37,141 Kb
State
2
1
2
1
2
[0-40]
H3K4me3
[0-40]
H3K9me3
[0-40]
H3K27me3
[0-150]
H3K27ac
[0-20]
H3K36me3
Marcks
h
Chr 12: 55,597-55,604 Kb
2
1
2
1
2
[0-40]
[0-40]
[0-40]
[0-40]
[0-10]
Insm2

Figure S4. Representative genes showing the pattern of occupancy in an open region, such as *Marcks*, and a bivalent region, such as *Insm2*, in the 1-month HFD (a, b), 3-month HFD (c, d), 6-month chow (e, f), and 6-month HFD datasets (g, h).

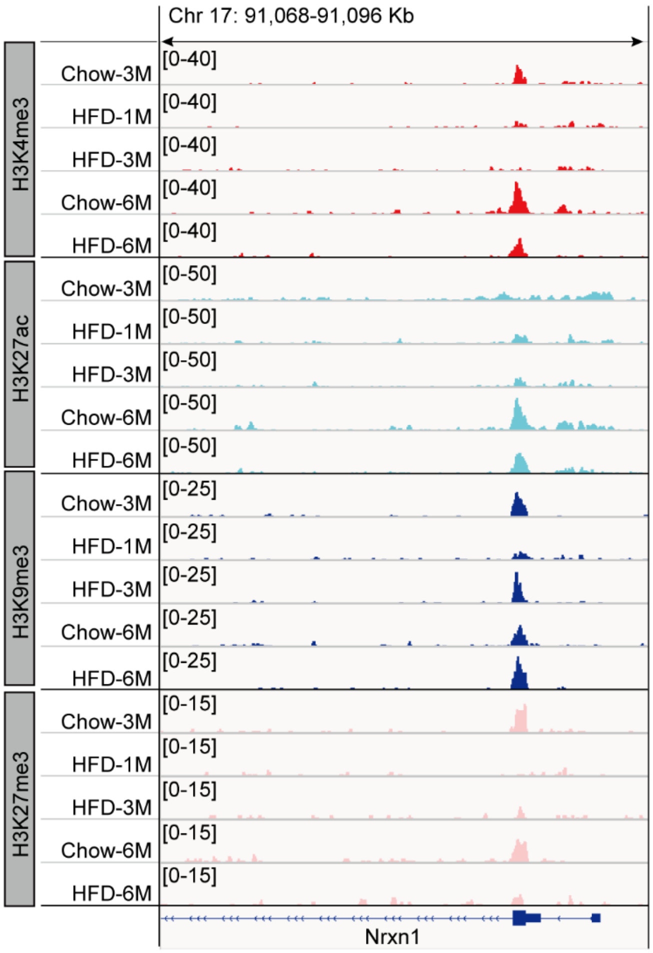

Figure S5. The pattern of histone modification of a representative gene, *Nrxn1*, in the five experimental groups.

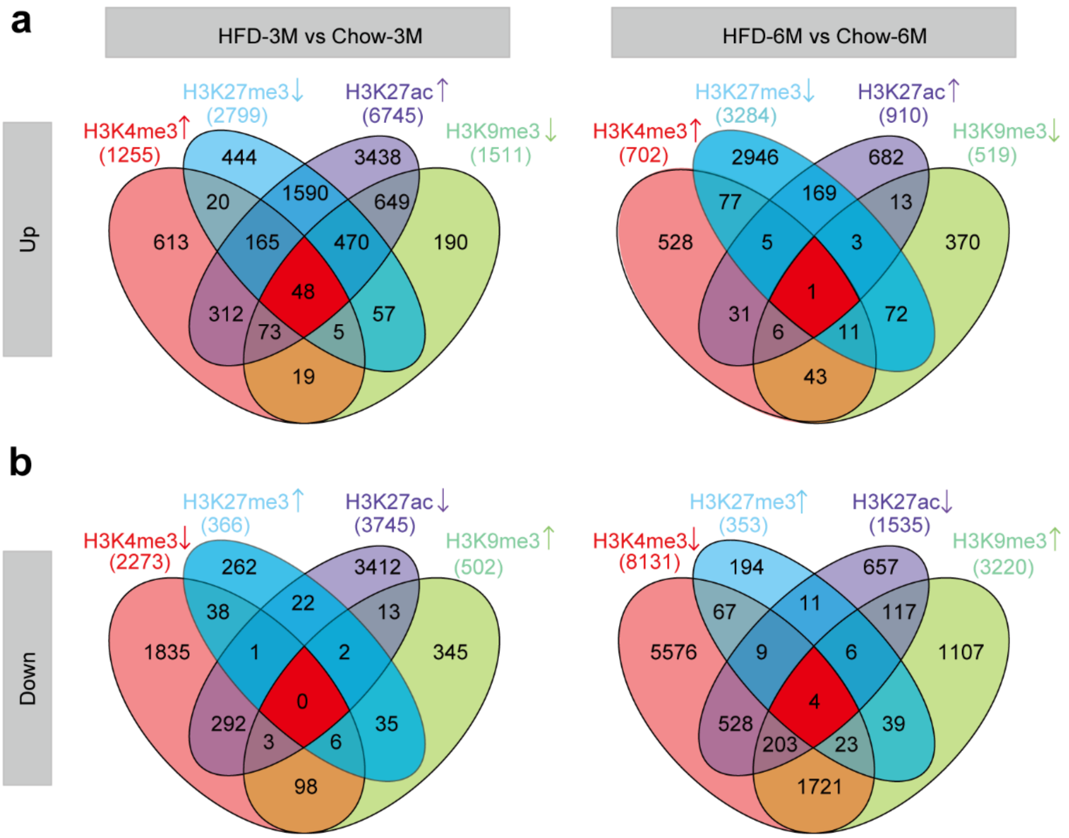

Figure S6. The commonly impacted genes inferred by ChIP-seq datasets in the indicated treatment groups. a) Venn diagram showing the number of imputed up-regulated genes whose promoter regions were enriched with H3K4me3 and H3K27ac, but not with H3K9me3 or H3K27me3 ChIP-seq peaks in mouse hypothalamus. b) Venn diagram showing the number of imputed down-regulated genes whose promoter regions were not enriched with H3K4me3 and H3K27ac, but with H3K9me3 or H3K27me3 ChIP-seq peaks in the hypothalamus.

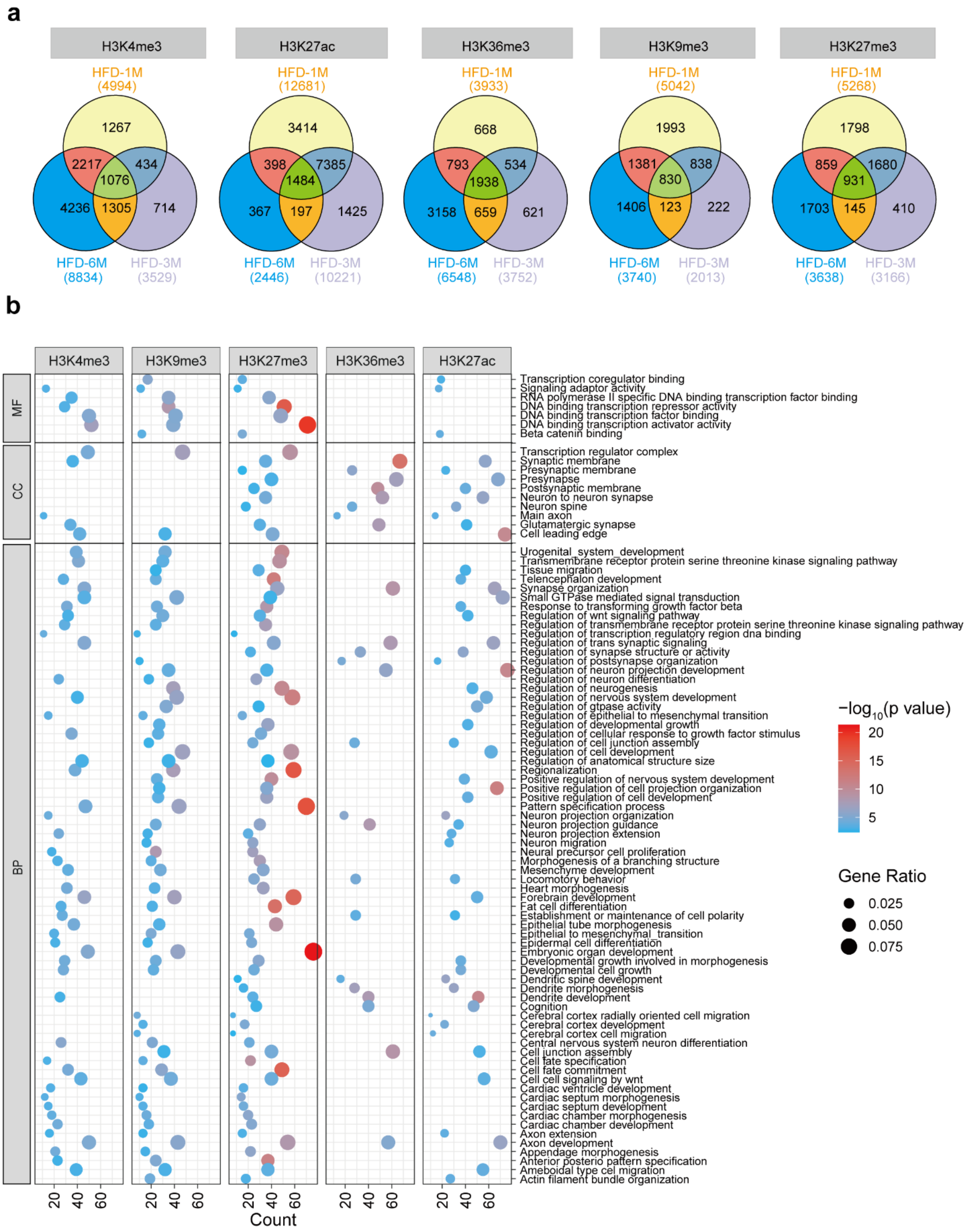

Figure S7. The overlapped genes whose promoter regions were differentially occupied by peaks of histone marks. a) Venn diagrams showing the overlapping of

genes whose promoter regions were differentially occupied by peaks from ChIP-seq of five histone marks. The gene body regions were also included for the H3K36me3 ChIP-seq data analysis. b) Dot plot showing significantly enriched GO terms for commonly impacted genes identified in panel a. The density of color represents the *p* value, and the dot size represents the gene ratio. MF, molecular function; CC, cellular component; BP, biological process.

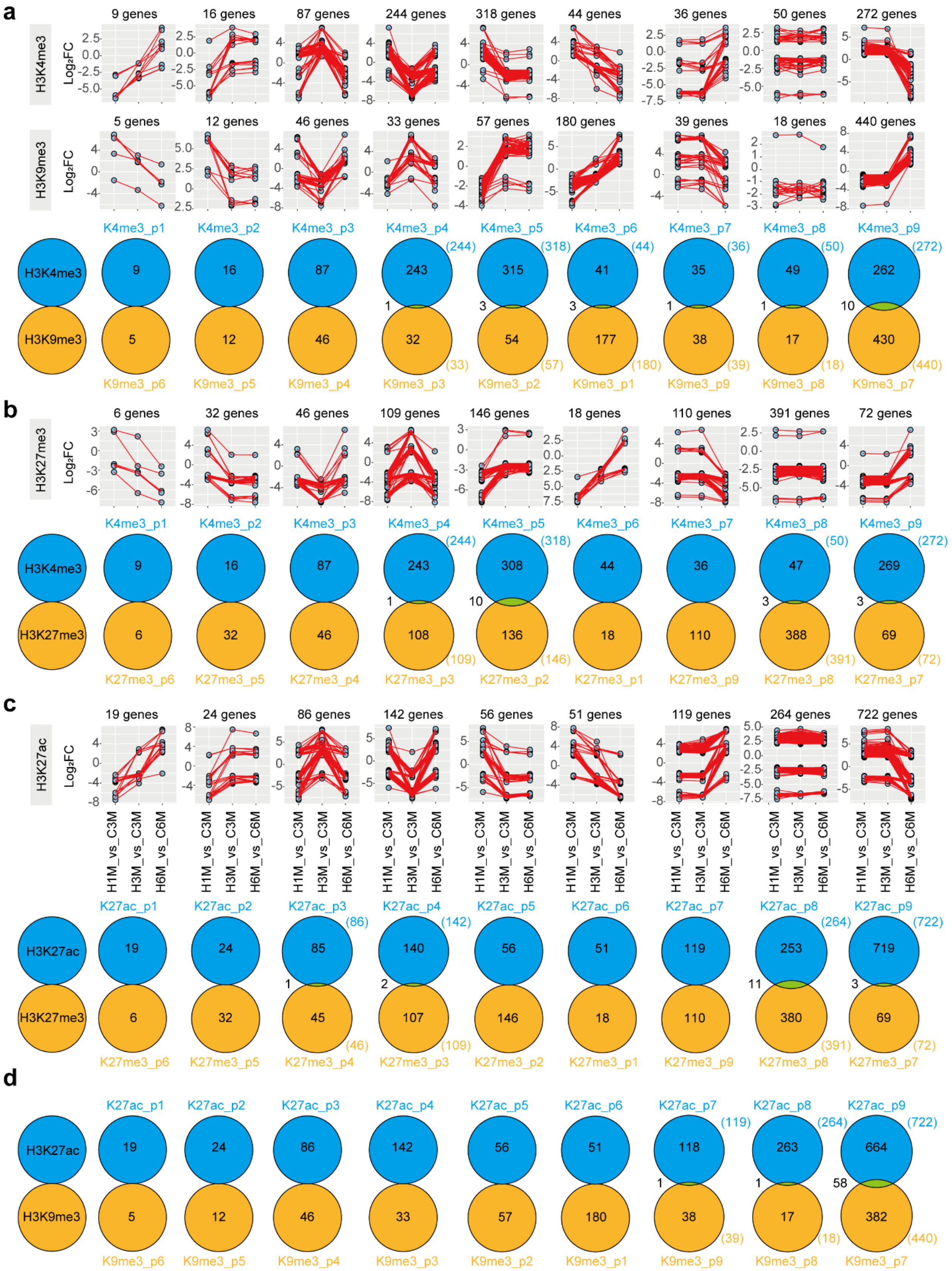

a
9 genes
16 genes
87 genes
244 genes
318 genes
44 genes
36 genes
50 genes
272 genes
H3K4me3
Log₂FC
5 genes
12 genes
46 genes
33 genes
57 genes
180 genes
39 genes
18 genes
440 genes
H3K9me3
Log₂FC
K4me3_p1
K4me3_p2
K4me3_p3
K4me3_p4
K4me3_p5
K4me3_p6
K4me3_p7
K4me3_p8
K4me3_p9
H3K4me3
9
16
87
243
(244)
315
(318)
41
(44)
35
(36)
49
(50)
262
(272)
1
3
3
1
1
10
H3K9me3
5
12
46
32
(33)
54
(57)
177
(180)
38
(39)
17
(18)
430
(440)
K9me3_p6
K9me3_p5
K9me3_p4
K9me3_p3
K9me3_p2
K9me3_p1
K9me3_p9
K9me3_p8
K9me3_p7
b
6 genes
32 genes
46 genes
109 genes
146 genes
18 genes
110 genes
391 genes
72 genes
H3K27me3
Log₂FC
K4me3_p1
K4me3_p2
K4me3_p3
K4me3_p4
K4me3_p5
K4me3_p6
K4me3_p7
K4me3_p8
K4me3_p9
H3K4me3
9
16
87
243
(244)
308
(318)
44
36
47
(50)
269
(272)
1
10
3
3
H3K27me3
6
32
46
108
(109)
136
(146)
18
110
388
(391)
69
(72)
K27me3_p6
K27me3_p5
K27me3_p4
K27me3_p3
K27me3_p2
K27me3_p1
K27me3_p9
K27me3_p8
K27me3_p7
c
19 genes
24 genes
86 genes
142 genes
56 genes
51 genes
119 genes
264 genes
722 genes
H3K27ac
Log₂FC
H1M_vs_C3M
H3M_vs_C3M
H6M_vs_C6M
K27ac_p1
K27ac_p2
K27ac_p3
K27ac_p4
K27ac_p5
K27ac_p6
K27ac_p7
K27ac_p8
K27ac_p9
H3K27ac
19
24
85
(86)
140
(142)
56
51
119
253
(264)
719
(722)
1
2
11
3
H3K27me3
6
32
45
(46)
107
(109)
146
18
110
380
(391)
69
(72)
K27me3_p6
K27me3_p5
K27me3_p4
K27me3_p3
K27me3_p2
K27me3_p1
K27me3_p9
K27me3_p8
K27me3_p7
d
K27ac_p1
K27ac_p2
K27ac_p3
K27ac_p4
K27ac_p5
K27ac_p6
K27ac_p7
K27ac_p8
K27ac_p9
H3K27ac
19
24
86
142
56
51
118
(119)
263
(264)
664
(722)
1
1
58
H3K9me3
5
12
46
33
57
180
38
(39)
17
(18)
382
(440)
K9me3_p6
K9me3_p5
K9me3_p4
K9me3_p3
K9me3_p2
K9me3_p1
K9me3_p9
K9me3_p8
K9me3_p7

Figure S8. Trend analysis of histone modification profile. The trend of changes of ChIP-seq peaks for the active histone modification mark H3K4me3 and suppressive histone mark H3K9me3 (a), H3K4me3 and H3K27me3 (b), H3K27ac and H3K27me3 (c), as well as H3K27ac and H3K9me3 (d). The numbers of genes impacted in both modifications are shown (a-d).

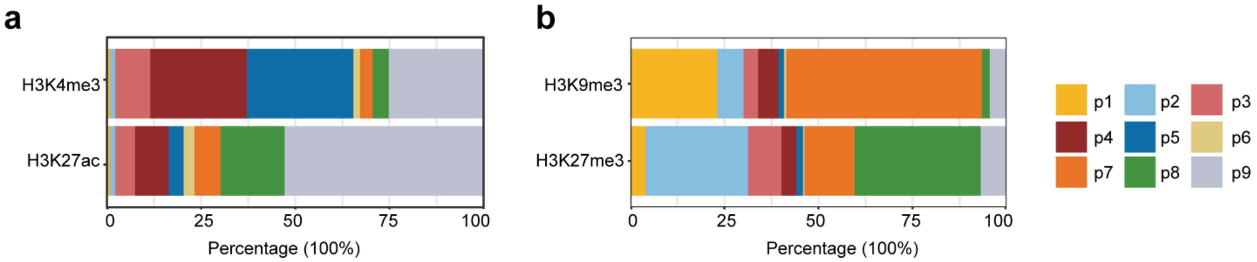

Figure S9. The proportion of profiles for H3K4me3 and H3K27ac (a), as well as H3K9me3 and H3K27me3 (b).

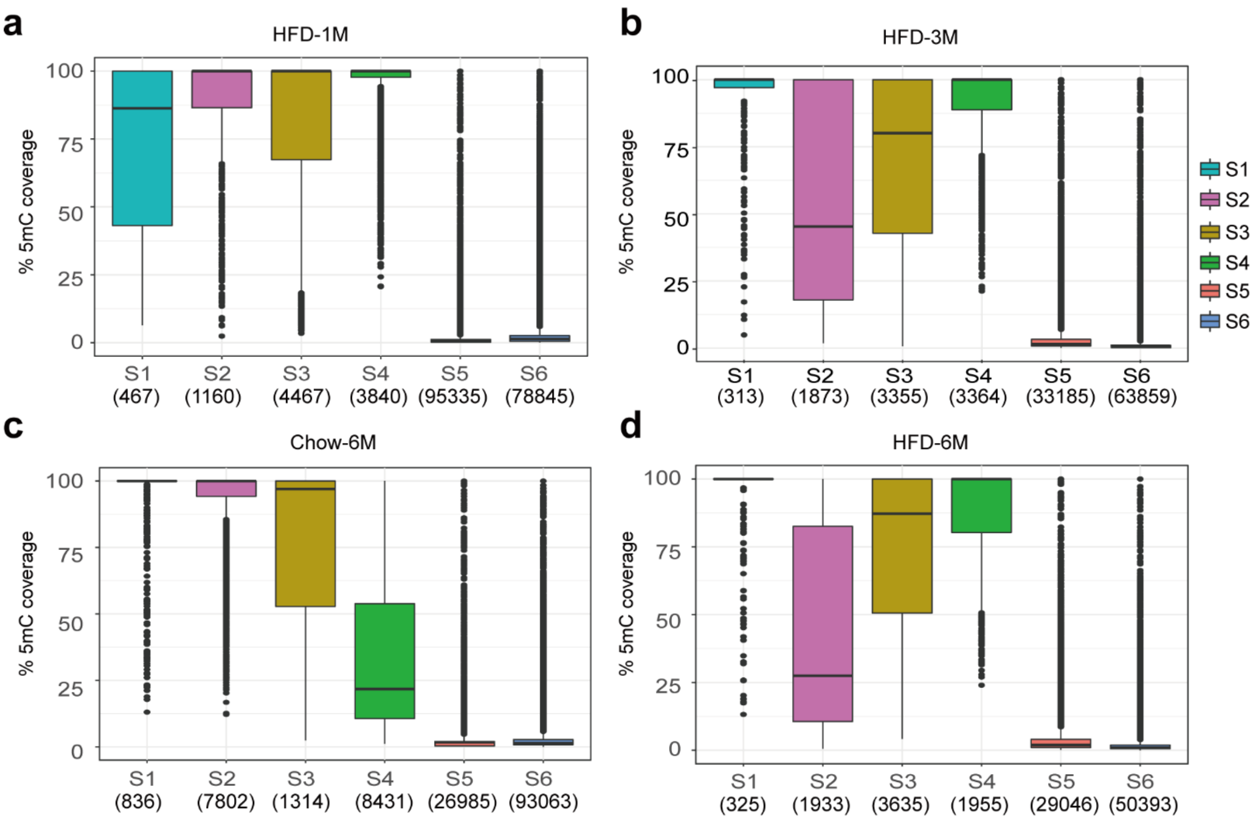

Figure S10. The DNA methylation level in each chromatin state. a-d) The DNA methylation levels in each chromatin state, which is the proportion of the peaks in MeDIP-seq over the genomic coverage of each state, are shown for the indicated treatment groups. The line in the boxplot represents the median and the boxes represent the first and third quartiles. The whiskers represent data that are within the 1.5× interquartile range. Data beyond the end of the whiskers are outlying points that are plotted individually. The numbers of overlapped regions between chromatin states and MeDIP-seq peak regions are shown at the bottom.

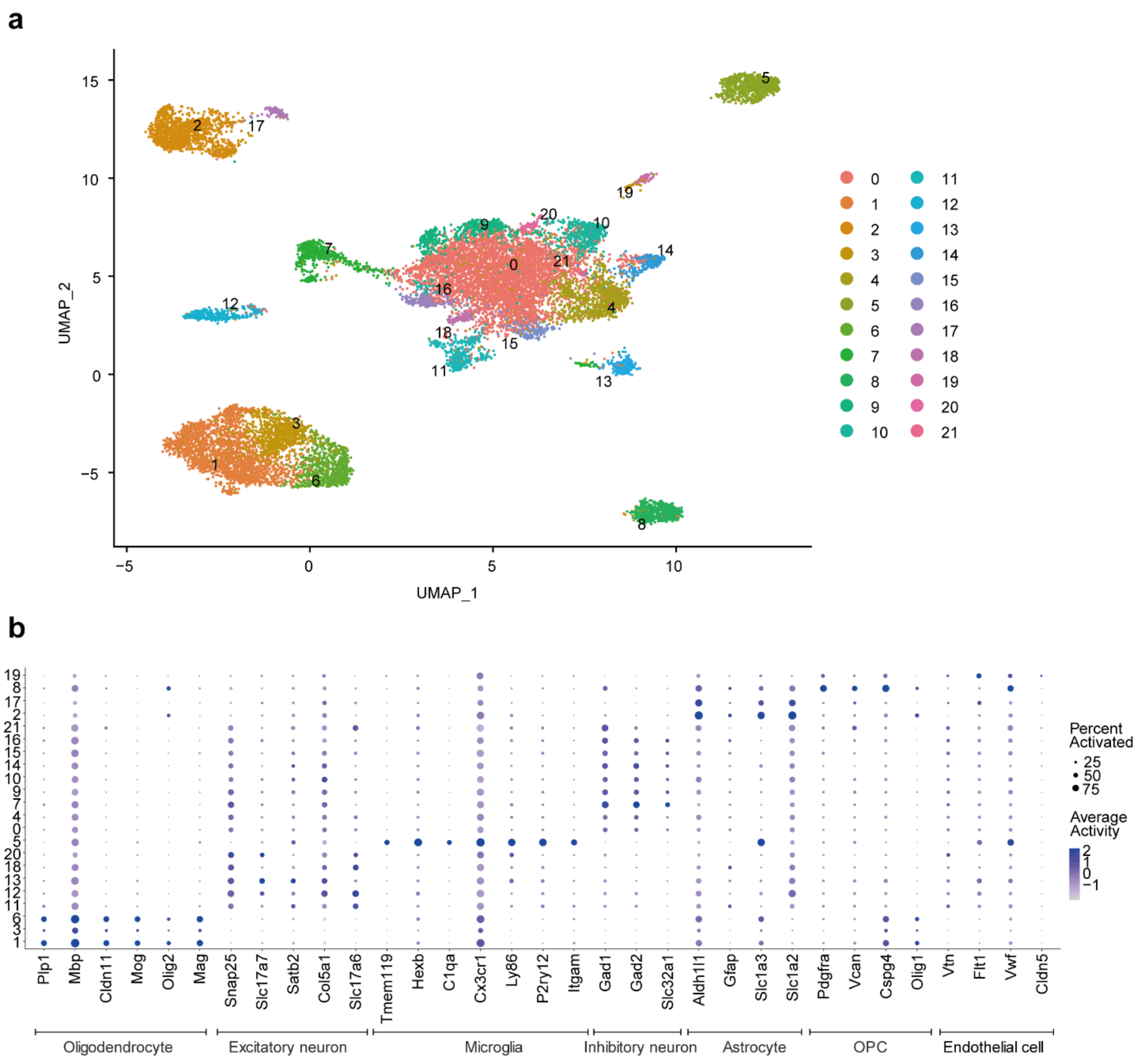

Figure S11. Unsupervised clustering of snATAC-seq data of mouse hypothalamus. a) Unsupervised clustering of the snATAC-seq dataset revealed 21 cell types. b) Dot plot showing the inferred marker gene activities in each cell type. Dot size corresponds to the proportion of cells with detected activity of the indicated gene, and dot density corresponds to average gene activity relative to that in all cell types.

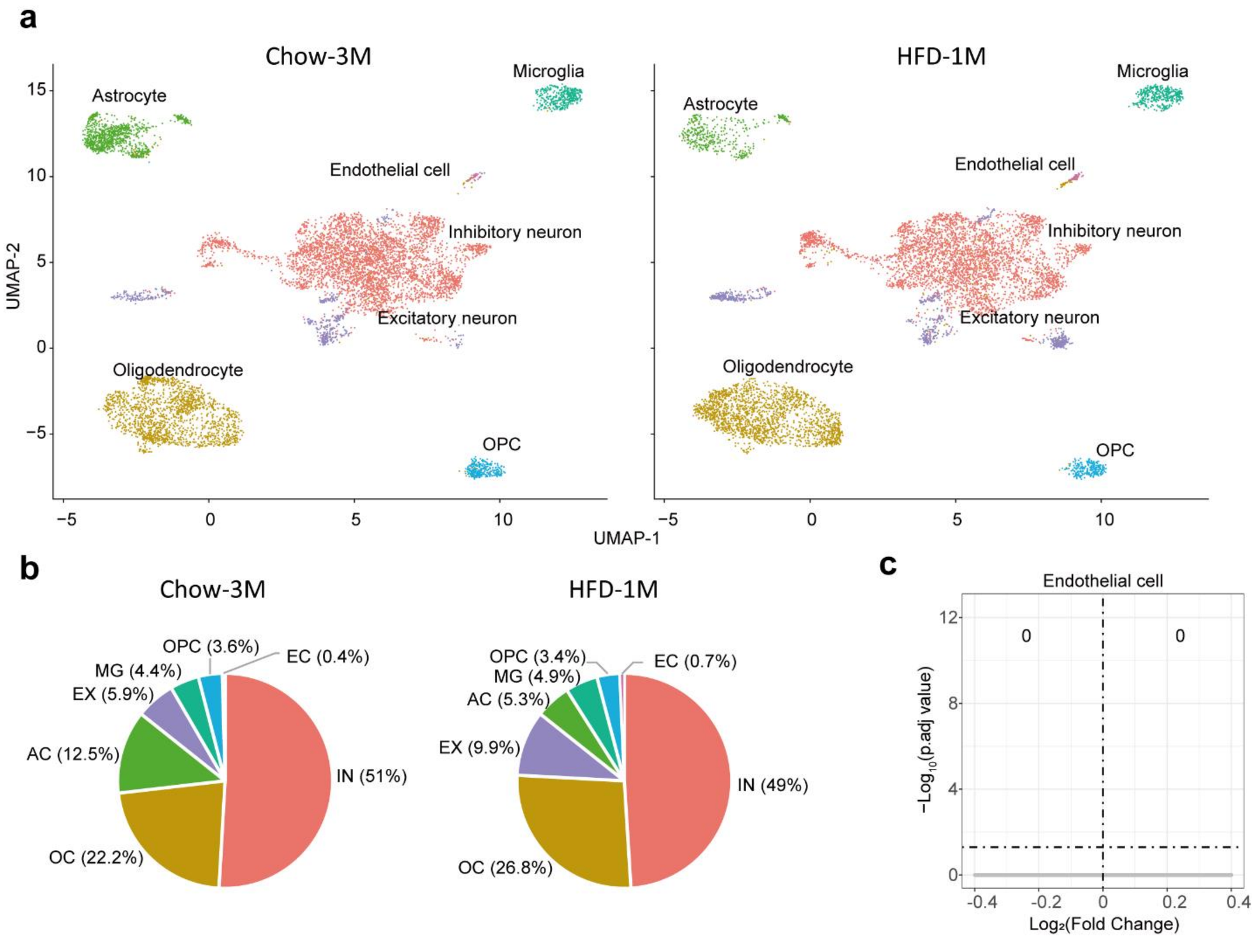

Figure S12. Major cell types in mouse hypothalamus were identified in snATAC-seq. a) UMAP plots of the snATAC-seq dataset. OPC, oligodendrocyte precursor cell; Chow-3M, chow-fed male C57 BL/6 mice; HFD-1M, age-matched C57 BL/6 mice were fed a HFD for 1 month. b) Cellular composition of the hypothalamus derived from the snATAC-seq data. EX, excitatory neuron; IN, inhibitory neuron; AC, astrocyte; MG, microglia; OC, oligodendrocyte; EC, endothelial cell. c) Volcano plots show genes with differential accessibilities in endothelial cells. The horizontal dashed lines indicate *p.adjust* = 0.05.

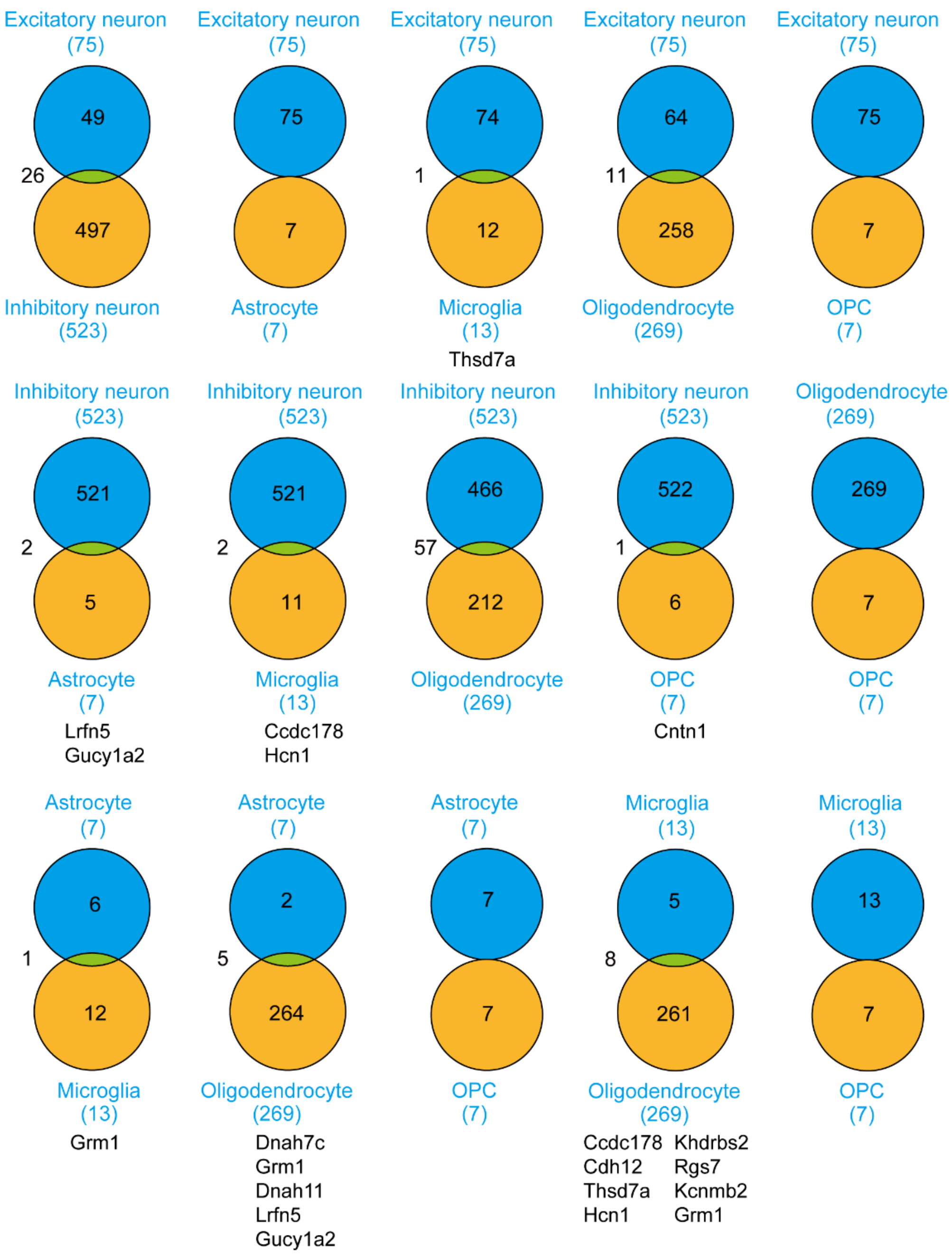

Figure S13. snATAC-seq data analysis to reveal the commonly impacted genes between two cell types. OPC, oligodendrocyte precursor cell.

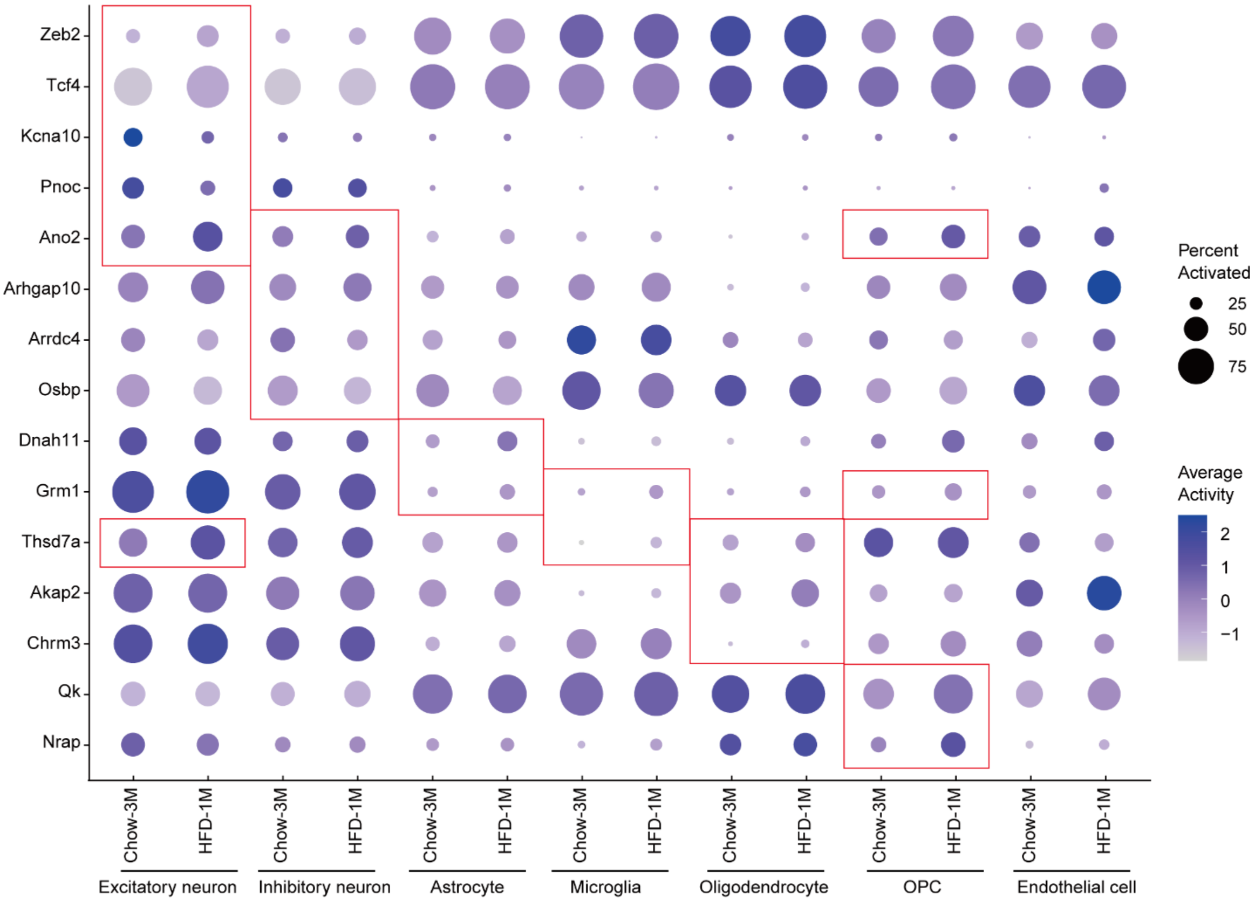

Figure S14. Representative genes impacted by HFD feeding in respective hypothalamic cell type. Red boxes indicate the significantly impacted genes between dietary treatment groups. Dot size corresponds to the proportion of cells with detected activity of the indicated gene and dot density corresponds to average gene activity relative to that in all cell types. OPC, oligodendrocyte precursor cell.

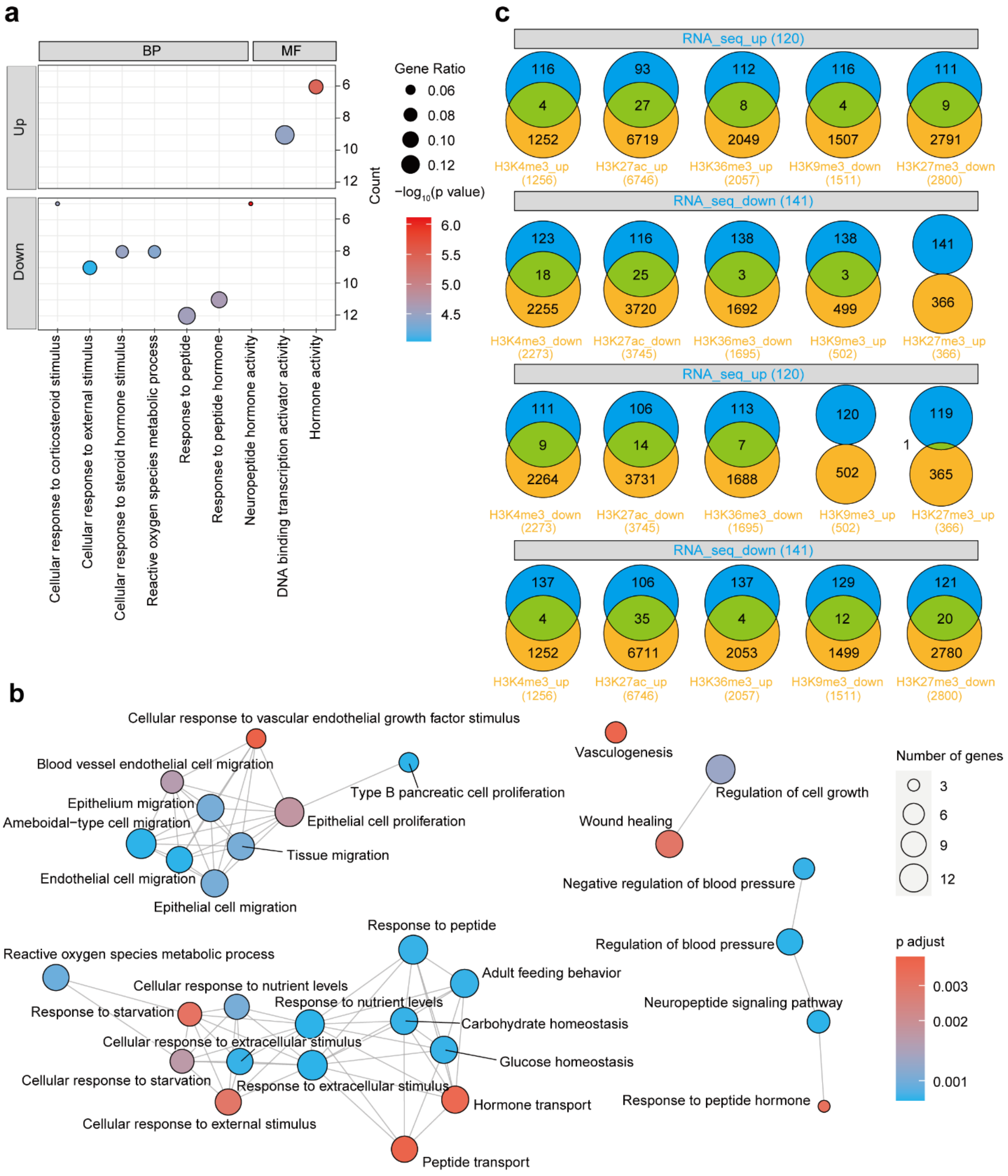

Figure S15. Integrated analysis of RNA-seq and ChIP-seq data. a) Dot plot showing significantly enriched GO terms for the up- or down-regulated genes. The color represents the *p* value, and the dot size represents the gene ratio. BP, biological process; MF, molecular function. b) Enrichment map networks displaying pathways for differentially

expressed genes in RNA-seq. Each node represents a gene set (i.e., a GO term), and each edge represents the overlap between two gene sets. The color represents the adjusted *p* value, and the dot size represents the gene number. c) Venn diagram showing the overlapping of the up- or down-regulated mRNAs with active and suppressive histone markers.
